# A-to-I RNA editing in bacteria increases pathogenicity and tolerance to oxidative stress

**DOI:** 10.1371/journal.ppat.1008740

**Published:** 2020-08-21

**Authors:** Wenhan Nie, Sai Wang, Rui He, Qin Xu, Peihong Wang, Yan Wu, Fang Tian, Junhua Yuan, Bo Zhu, Gongyou Chen

**Affiliations:** 1 Key Laboratory of Urban Agriculture by Ministry of Agriculture of China, School of Agriculture and Biology, Shanghai Jiao Tong University, Shanghai, China; 2 Hefei National Laboratory for Physical Sciences at the Microscale and Department of Physics, University of Science and Technology of China, Hefei, Anhui, China; 3 State Key Laboratory of Microbial Metabolism, and SJTU-Yale Joint Center for Biostatistics and Data Science, School of Life Sciences and Biotechnology, Shanghai Jiao Tong University, Shanghai, China; 4 State Key Laboratory for Biology of Plant Diseases and Insect Pests, Institute of Plant Protection, Chinese Academy of Agricultural Sciences, Beijing, China; University of Missouri, UNITED STATES

## Abstract

Adenosine-to-inosine (A-to-I) RNA editing is an important posttranscriptional event in eukaryotes; however, many features remain largely unexplored in prokaryotes. This study focuses on a serine-to-proline recoding event (S128P) that originated in the mRNA of *fliC*, which encodes a flagellar filament protein; the editing event was observed in RNA-seq samples exposed to oxidative stress. Using Sanger sequencing, we show that the S128P editing event is induced by H_2_O_2_. To investigate the *in vivo* interaction between RNAs and TadA, which is the principal enzyme for A-to-I editing, genome-wide RNA immunoprecipitation–coupled high-throughput sequencing (iRIP-Seq) analysis was performed using HA-tagged TadA from *Xanthomonas oryzae* pv. *oryzicola*. We found that TadA can bind to the mRNA of *fliC* and the binding motif is identical to that previously reported by Bar-Yaacov and colleagues. This editing event increased motility and enhanced tolerance to oxidative stress due to changes in flagellar filament structure, which was modelled in 3D and measured by TEM. The change in filament structure due to the S128P mutant increased biofilm formation, which was measured by the 3D laser scanning confocal microscopy. RNA-seq revealed that a gene cluster that contributes to siderophore biosynthesis and Fe^3+^ uptake was upregulated in S128P compared with WT. Based on intracellular levels of reactive oxygen species and an oxidative stress survival assay, we found that this gene cluster can contribute to the reduction of the Fenton reaction and increases biofilm formation and bacterial virulence. This oxidative stress response was also confirmed in *Pseudomonas putida*. Overall, our work demonstrates that A-to-I RNA editing plays a role in bacterial pathogenicity and adaptation to oxidative stress.

## Author summary

Adenosine-to-inosine (A-to-I) RNA editing is an important posttranscriptional event in eukaryotes that has only been recently documented in bacteria. In this study, we use multiple ‘omic’ approaches to show that A-to-I RNA editing can occur in *fliC*, a flagellar filament protein. We show that TadA, which encodes adenosine deaminase, can directly bind to mRNA of target genes through recognition of a GACG motif. This editing event changes a single amino acid residue from serine to proline in FliC, resulting in a structural change in the flagellar filament. This posttranscriptional editing event contributes to virulence and increases tolerance to oxidative stress by enhancing biofilm formation. Our results provide insight into a new mechanism that bacterial pathogens use to adapt to oxidative stress, which can also increase virulence.

## Introduction

RNA editing is a posttranscriptional event that alters genomic RNA sequences via insertion, deletion, deamination or substitution [1, 2]. Adenosine-to-inosine (A-to-I) editing, which is catalyzed by the adenosine deaminase RNA-specific (ADAR) family of enzymes, is the most prevalent type of editing in metazoans [3, 4]. This form of editing functions in multiple biological processes ranging from nonsense-mediated mRNA decay and alternative splicing to gene expression and translation [5, 6]. Since inosine (I) is recognized as guanosine (G) by the polymerase enzyme and translational machinery, A-to-I editing in coding regions of the mRNA may lead to codon changes, resulting in further diversification of protein function [2]. The importance of A-to-I editing in eukaryotes has been confirmed and occurs in both noncoding [3, 7] and coding regions [8, 9]. Nevertheless, in prokaryotes, A-to-I RNA editing of mRNA has rarely been reported.

The development of sequencing technologies has led to the discovery and identification of RNA editing and recoded sites in metazoans [10–14] and fungi [8, 15–17]. Recently, Bar-Yaacov and colleagues demonstrated that nonsynonymous A-to-I editing can occur in bacterial mRNA [18]. The authors found that A-to-I editing in *hokB*, which encodes a toxin that confers antibiotic tolerance, increases as a function of cell density and enhances toxicity of the protein [18]. Furthermore, tRNA-specific adenosine deaminase (TadA) was identified as the A-to-I editing enzyme in *Escherichia coli*, which expands the role of *tadA* as both a tRNA- and mRNA-editing enzyme in bacteria [19]. However, it remains unclear whether A-to-I editing is a universal mechanism for posttranscriptional modification in bacteria. Additionally, it is unclear whether bacterial growth, life cycle, pathogenicity and stress responses are regulated by A-to-I editing.

In this study, we developed an in-house Python script for detection of A-to-I editing events in bacteria. By applying this script, we profiled and identified A-to-I editing events in *Xanthomonas oryzae* pv. *oryzicola* (*Xoc*) BLS256 during oxidative stress. After filtering out the synonymous editing sites, we identified a serine to proline mutation (S128P) in the flagellar filament protein FliC after bacteria were exposed to H_2_O_2_. In this study, we identified two editing events that resulted in nonsynonymous mutations in *Xoc* during oxidative stress. We then systematically analyzed one editing event and demonstrate its significance in response to stress and bacterial virulence.

## Results

### A-to-I detection pipeline development

The A-to-I modification analysis pipeline (AIMAP; S1 Fig) is available at https://github.com/castualwang/aimap. The pipeline was first tested on published A-to-I bacterial RNA-seq data [18] on a desktop machine equipped with an Intel (R) Xeon (R) CPU X5675 x12 processor, 64 GB RAM, and the Ubuntu 16.04 operating system. The total run time was approximately 80 min, and results are shown in S1 Table. All previously reported A-to-I editing sites were detected by AIMAP, with slightly different coverage due to the strict raw read processing requirement of our pipeline.

### Identification and functional prediction of A-to-I editing events

Potential A-to-I editing sites were identified based on our previous data from deep-sequenced *Xoc* BLS 256 cells in the presence or absence of 0.1 mM H_2_O_2_ [20]. Editing events were filtered based on the following criteria: (1) editing sites should be in the coding region and cause nonsynonymous mutations; (2) the frequency of editing site should be higher than 3% and supported by two biological replicates to remove possible errors; and (3) editing sites should exist at different time points after H_2_O_2_ exposure. After filtering, A-to-I editing events were identified in the coding regions of *XOC_2374* and *XOC_3486*, which encode flagellar protein FliC [21] and ferric enterobacter receptor (S2 Table). The PROVEAN scores of these two mutations were -2.2 and 0.8 for XOC_2374 and XOC_3486, respectively (S2 Table).

**Fig 1:**
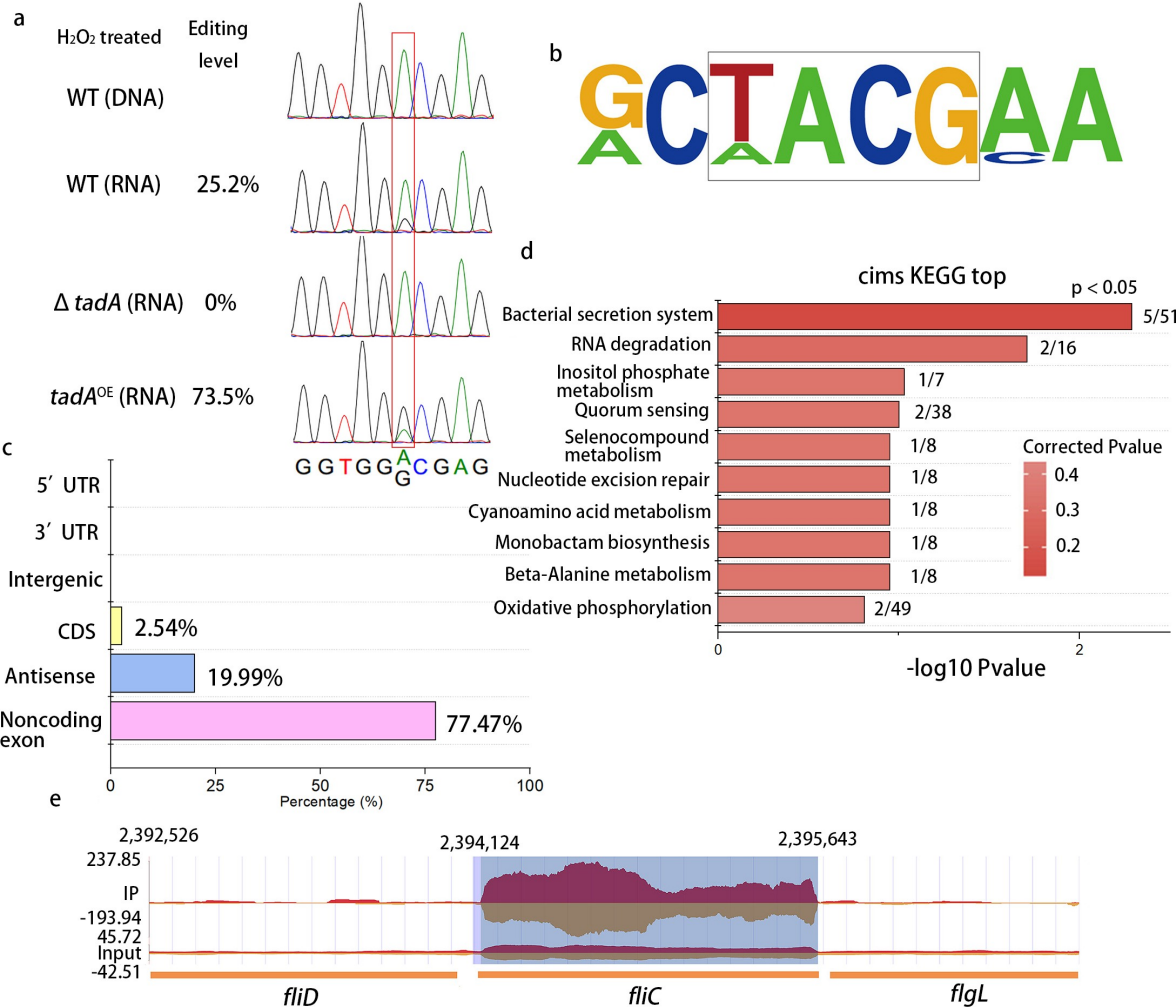
Analysis of the *fliC* (S128P) A-to-I RNA editing event by Sanger sequencing, RNA-seq and iRIP-seq. (a) Sequence analysis of DNA (gDNA) and/or cDNA in edited regions of wild-type (WT), *ΔtadA*, and *tadA*^*o*e^. PCR fragments were obtained from genomic DNA or cDNA (mRNA) using primers described in Methods. Samples were exposed to 0.1 mM H_2_O_2_ and then subjected to Sanger sequencing. Chromatograms show edited sites in gDNA and cDNA, and the percentage of edited sequence as determined by pyrosequencing is indicated. (b) Conserved motif in A-to-I edited sites based on RNA-seq. All sites share a conserved TACG motif that is identical to the four nucleotide sequence recognized by TadA. (c) Percentage of reads used for peak calling in iRIP-seq. (d) KEGG analysis of genes that were enriched in iRIP-seq. (e) Peaks that were called at *fliC* in the iRIP-seq data.

To exclude errors introduced by RNA-seq or in the published genome sequence, the editing in *fliC* was verified by Sanger sequencing (Fig 1A). Genomic DNA was sequenced and found as expected with no editing. cDNA was generated by RT-PCR from the wild-type grown in 0.1 mM H_2_O_2_ for 15 min, and directly sequenced by Sanger sequencing. The A-to-G change was confirmed in RNA samples as shown in Fig 1A. In contrast to the WT, no base changes were observed in *ΔtadA* cDNA. Furthermore, the percentage of base change was extremely high in *tadA*^*OE*^ cDNA, verifying the importance of *tadA* in A-to-I editing.

### The S128P editing event in *fliC* is induced by H_2_O_2_

The A-to-I editing event in *fliC* resulted in an amino acid change from serine to proline at residue 128 (S2 Table); this mutant was subsequently designated as S128P (S5 Table). The S128P editing event only occurred in *Xoc* samples exposed to H_2_O; thus we hypothesized that the *fliC* S128P mutation was induced by oxidative stress.

In *E*. *coli*, the *tadA* gene encoding tRNA-specific adenosine deaminase was responsible for bacterial tRNA and mRNA editing [18]. To investigate this possibility in *Xoc*, we constructed the *tadA* deletion and overexpressing strains, *ΔtadA* and *tadA*^*OE*^, respectively (see Methods). We then analyzed potential editing events in gDNA and cDNA samples from WT, Δ*tadA*, and *tadA*^OE^ in the presence and absence of 0.1 mM H_2_O_2_ (Fig 1A). In the presence of H_2_O_2,_ 25.5% of WT RNA samples showed editing events; however, no editing was detected in cDNA samples of *ΔtadA* (Fig 1A). In the *tadA*^OE^ cDNA sample exposed to H_2_O_2_, 73.5% of the cDNA samples exhibited an editing event (Fig 1A). These results suggest that the serine to proline editing event is induced by H_2_O_2_ and modulated by *tadA*.

### iRIP-seq confirms the direct binding of TadA to *fliC* mRNA

iRIP-seq analysis was conducted to evaluate whether TadA binds to *fliC* mRNA. In this experiment, RNA/proteins from *Xoc* His-BLS256 were UV-cross-linked and RNA-bound proteins were immunoprecipitated with His-Tag antisera, which recognizes an engineered epitope in TadA of His-BLS256 (S5 Table). This was followed by MNase treatment and RNA extraction for paired-end deep sequencing using the Illumina NextSeq 500 system. A total of 45 million sequencing reads were obtained and mapped. Peak-calling analysis was then performed on the iRIP-seq datasets to identify TadA interaction partners. We found that most of the peaks were located at tRNA regions (Fig 1C), since the main function of this protein is deamination of tRNA [19]. In total, we found 30 enriched RNAs from coding genes in the TadA iRIP-Seq datasets. Interestingly, KEGG analysis revealed that the bacterial secretion system was the only enriched pathway (Fig 1D, *P* < 0.05), and this enriched set of genes included *fliC* (Fig 1E). Furthermore, peaks were enriched in the *fliC* gene (Fig 1E), which is consistent with previous findings in *E*. *coli* [18]. Overall, our data suggest that the TadA interaction with *fliC*-mRNA is significant.

### *Xoc* S128P and *P*. *putida* S491P mutants show increased tolerance to oxidative stress

**Fig 2:**
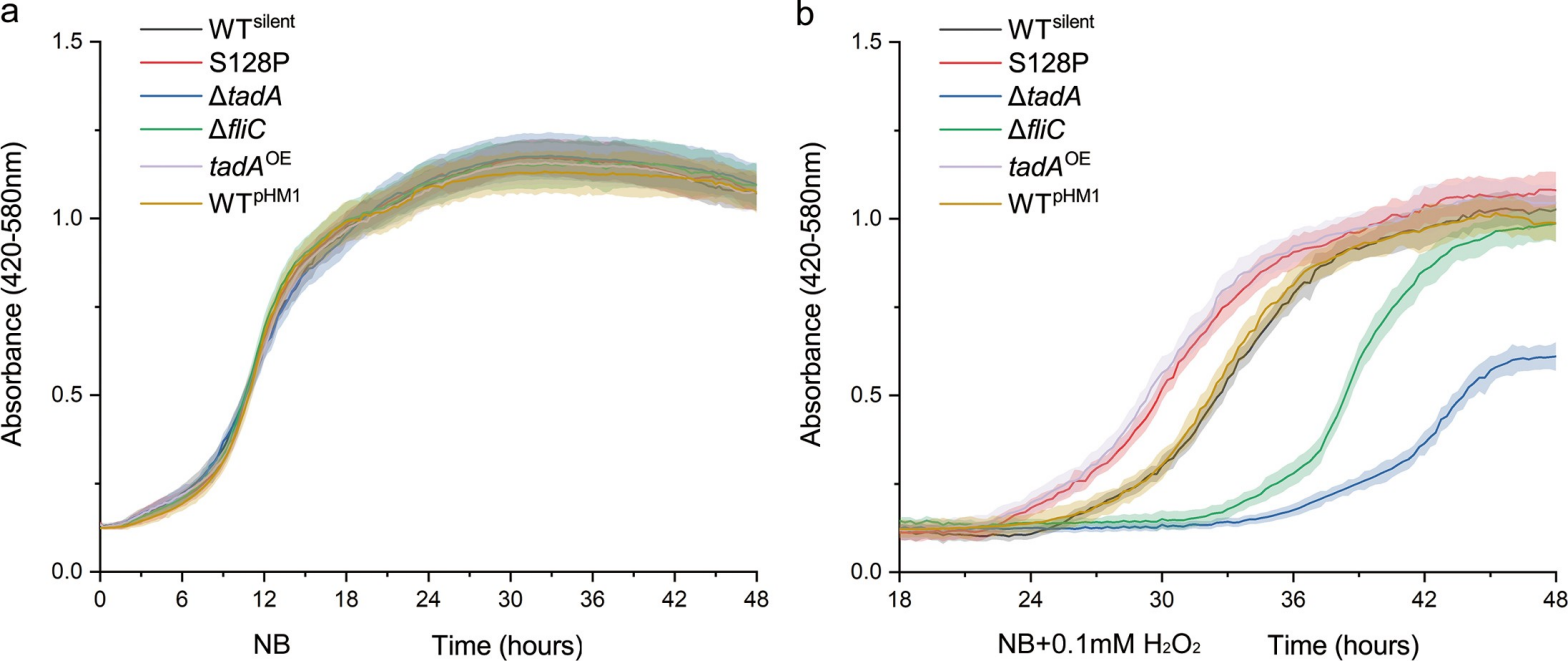
Growth of *Xoc* WT^silent^, S128P, Δ*tadA*, Δ*fliC*, *tadA*^OE^ and WT^pHM1^ in NB medium. Strains were grown in quadruplicate to mid-exponential phase, diluted to OD_600_ = 0.1, transferred to fresh NB and placed in a Bioscreen C apparatus at 28°C. Panels: (a) growth in NB; (b) growth in NB supplemented with 0.1 mM H_2_O_2_. Error intervals (shaded regions) indicate mean ± SE of *n* = 4 replicates.

Since the S128P editing event was induced by H_2_O_2_, we speculated that editing may function in oxidative stress. To further investigate, growth of selected strains was compared in the presence and absence of 0.1 mM H_2_O_2_ in NB medium (Fig 2). Strains grown in NB medium without H_2_O_2_ showed similar growth patterns (Fig 2A); however, a delayed lag phase of approximately seven hours was observed in the S128P and *tadA*^OE^ strains grown in NB supplemented with 0.1 mM H_2_O_2_. Pairwise comparisons of OD values for each strain and growth condition were analyzed with the Kolmogorov-Smirnov test against the values obtained for WT^silent^. The S128P and *tadA*^OE^ strains grown in 0.1 mM H_2_O_2_ showed enhanced tolerance (*P*<0.01), while Δ*tadA* showed a significantly lower tolerance to oxidative stress when compared with WT^silent^ (*P*<0.01, Fig 2B). These differences were substantiated by viable plate counts (S2 Fig). These results strongly suggest that the S128P editing event helps *Xoc* adapt to oxidative stress.

To evaluate the significance of the above results, we generated a serine to proline mutation in amino acid residue 491 of *P*. *putida* FliC (see Methods). *In vitro* growth curves revealed that the S491P mutant was more tolerant to H_2_O_2_ than the wild-type KT2440 (S10 Fig, *P*<0.01). These results indicate that posttranscriptional A-to-I modifications may be widespread in bacteria undergoing oxidative stress.

### S128P editing impacts the structure of flagellar filaments

**Fig 3:**
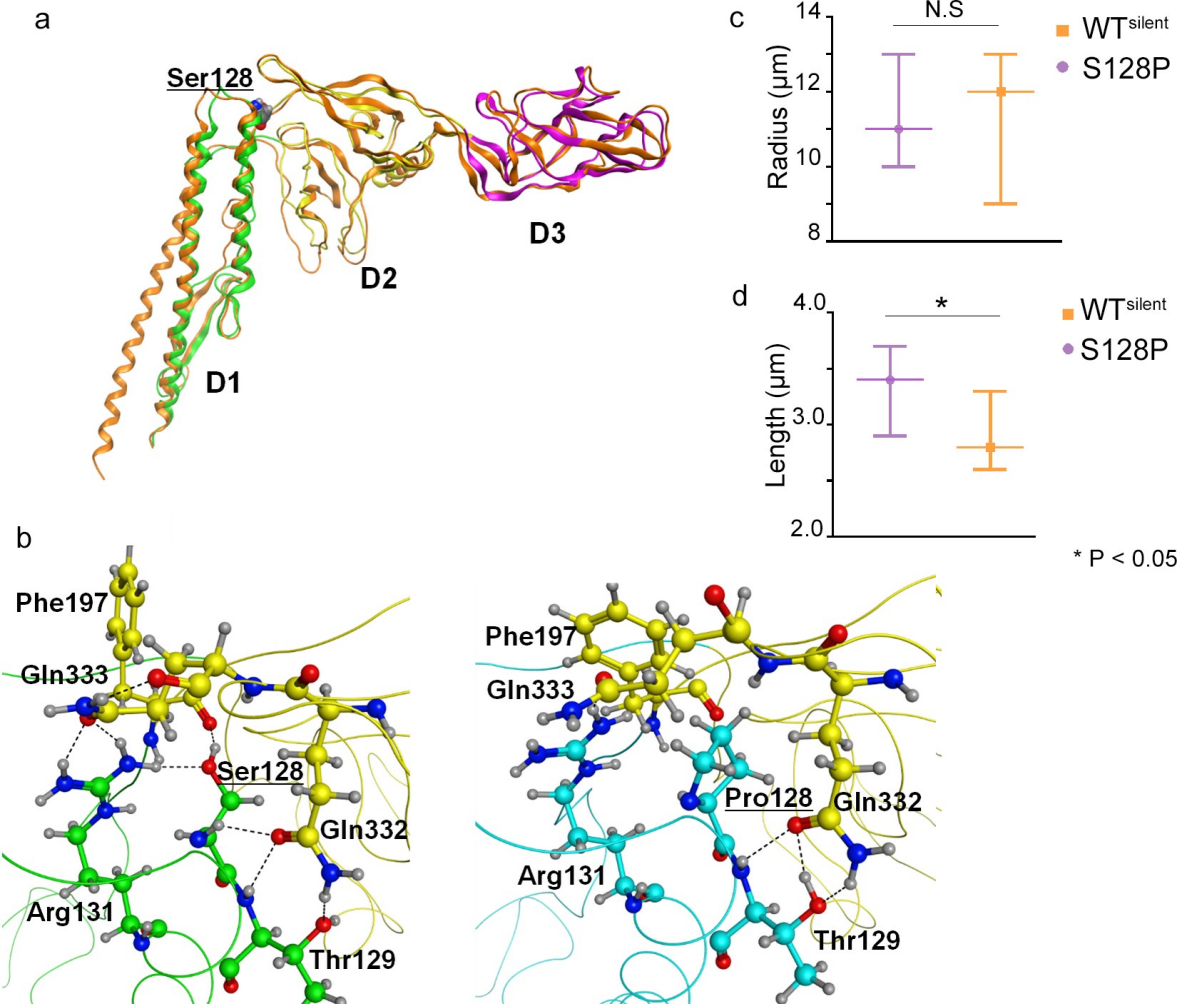
Homology modeling of FliC and measurement of flagellar filament length and radius. (A) Homology model of FliC using *P*. *aeruginosa* flagellin 4NX9 (PDB ID: 1IO1) as the template. The backbone is colored green, yellow and magenta at the D1, D2 and D3 domains of the model, respectively, and with orange for the template. The serine residue (Ser128) is depicted using spheres. (B) Comparison of hydrogen bonding around the mutation site. The loss of several critical H bonds in the Ser128Pro mutant (right) may disrupt the H-bonding network surrounding Ser128 (left), which stabilizes the D1-D2 interface. The carbons of the D1 residues are colored green in the WT model (left) and with cyan in the mutant (right), whereas D2 residues are colored yellow in both models. Measurement of filament length (C) and radii (D) from 120 cells of WT^silent^ and the S128P mutant. Error bars show the 95% CI around the mean for each sample. Statistically significant differences occurred when the mean of one sample did not lie within the 95% CI.

The FliC protein is the primary flagellar filament functioning in bacterial motility [22]; furthermore, motility is positively correlated with the formation and architecture of bacterial biofilms [23]. Thus, we hypothesize that the S128P editing event could change flagellar filament structure and thereby result in altered motility and biofilm formation. To further investigate the impact of the S128P exchange, 3D structures of FliC were predicted (Fig 3A). We found that S128P is located at the beginning of an α-helix; furthermore, the edited residue was predicted to reside in a loop within the mRNA secondary structure (S3 Fig). A homology model of WT FliC was constructed based on the X-ray structure of *Pseudomonas aeruginosa* flagellin (PDB ID, 1IO1). Sequence alignment and the superimposed backbones of the homology model and template suggest that their similarity is reliable, especially at the D1-D2 interface surrounding the mutation site (Fig 3A). When Ser128 underwent mutation to proline, modeling suggests a minor deviation in the backbone of the loop where the mutation site resides (Fig 3B). More importantly, the H bonds at Ser128, which may help stabilize the D1-D2 interface, are lost in the Ser128Pro mutant. In WT FliC, Ser128 forms H bonds with the backbone of Phe197 and the sidechains of Arg131 and Gln332 (Fig 3B). The relatively fixed sidechains of Arg131 and Gln332 can form H bonds with Gln333 and Thr129, respectively, to further stabilize the D1-D2 interface. However, the H-bonding network is disrupted in the Ser128Pro mutant. Although the sidechains Arg131 and Gln332 can still form H bonds with Gln333 and Thr129, respectively, they are not fixed to the core residue Pro128.

The loss of H bonds could reduce the interaction between the D1 domain and hypervariable region of FliC (Fig 3B), which was proposed to have bacterial adhesin-like properties [24]. Based on that, we infer that the S128P mutation may cause structural changes in the flagellar filament. To test this hypothesis, characteristics of the flagellar filament including the radius and length were measured by TEM. We observed significantly longer filament lengths (*L*) for WT^silent^ as compared to the S128P mutant (Fig 3D), suggesting that the Ser-to-Pro mutation impacts filament structure. However, there were no significant differences in the radii of filaments (Fig 3C).

### S128P editing and bacterial motility

The flagellar filament is essential for motility; thus, we speculated that S128P editing may impact motility. We used the equation described in Methods to calculate motility (*F*). Since S128P editing changes filament structure, filament length (*L*, increase, confirmed above) and swimming speed (*ν*, not confirmed) should be the only altered parameters. To test whether swimming speed is modified, direct measurements were performed using phase contrast microscopy. We calculated the mean value of each distribution, and the mean speeds were 30.17 and 29.89 μm/s for the WT^silent^ strain and S128P strain, respectively (S4 Fig and S1 Video). This result suggests there is no significant difference in swimming speed (*ν*) between WT^silent^ and S128P. However, filament length (*l*) was longer in S128P relative to WT^silent^ (Fig 3D). As described in equation, motility ability (*F*) is positively influenced by *ν* and *l*. Thus, based on the equation, we concluded that the motility of S128P is higher than WT^silent^.

### S128P editing enhances bacterial biofilm formation

**Fig 4:**
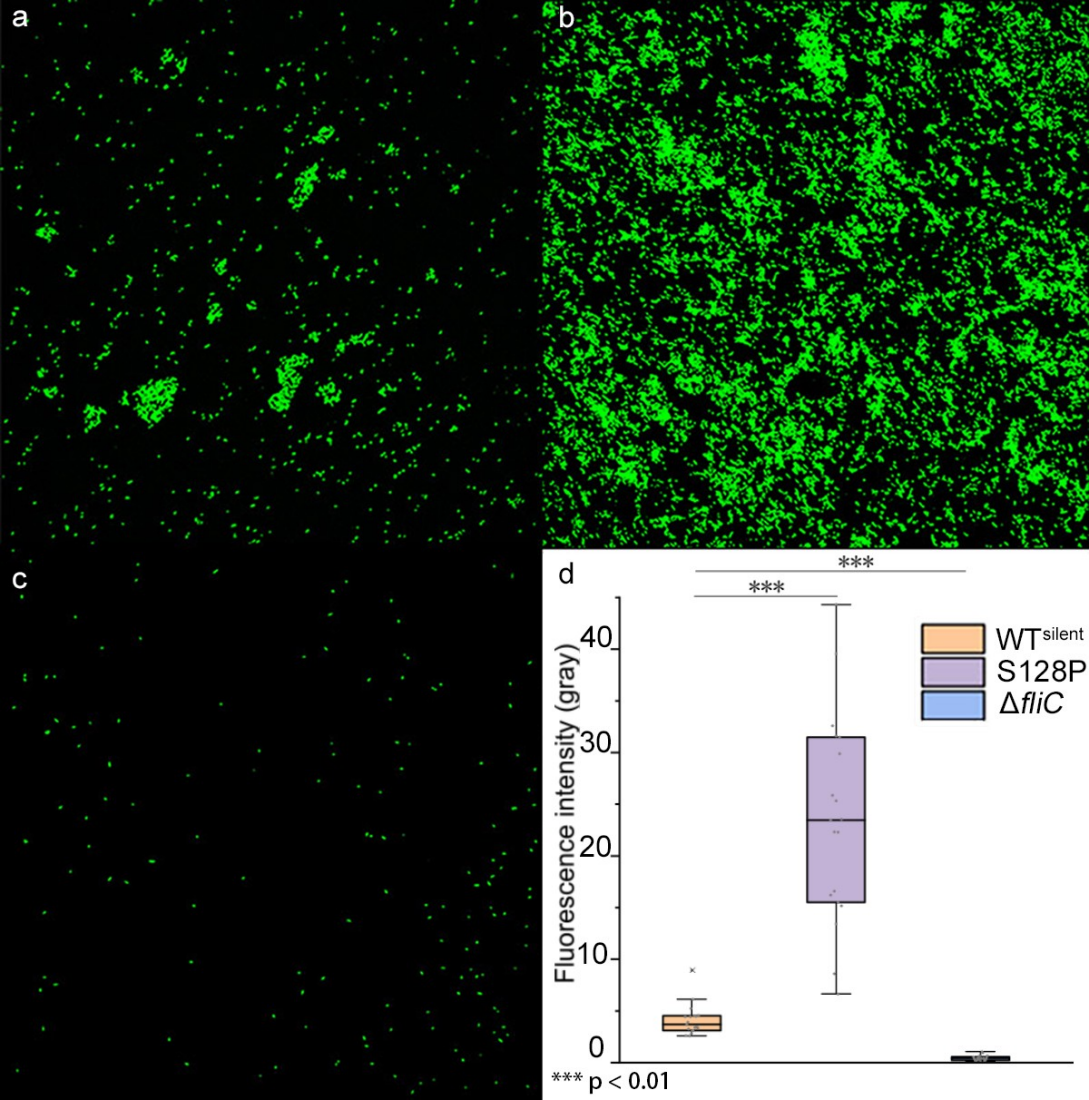
Confocal laser scanning microscopy of fluorescence in GFP-labeled (a) *Xoc* WT^silent^, (b) S128P mutant, and (c) Δ*fliC* mutant. Panel (d) shows fluorescence intensity in GFP-labeled *Xoc* WT^silent^, S128P, and Δ*fliC*. Bacteria were grown under static conditions at 28°C for 96 h on glass coverslips. Biofilms were fixed, and fluorescence intensity was randomly measured from 12 parallel replicates. ***, *P*<0.001, Wilcoxon rank-sum test.

Biofilm formation plays a pivotal role in the response of bacteria to oxidative stress [25, 26]; thus, we measured biofilm formation by 3D serial layer scanning using CLSM. Mutant S128P exhibited higher levels of adhesion to glass surfaces than WT^silent^, whereas Δ*fliC* was severely inhibited in its ability to adhere (Fig 4, S2 Video). These results are consistent with previous reports [21, 27]. When *Xoc* biofilms were observed by SEM, aggregated cocci and extracellular matrix-like substances were clearly observed for the mutant S128P (S5 Fig), which is consistent with results obtained with CLSM.

### S128P mutation contributes to virulence

**Fig 5:**
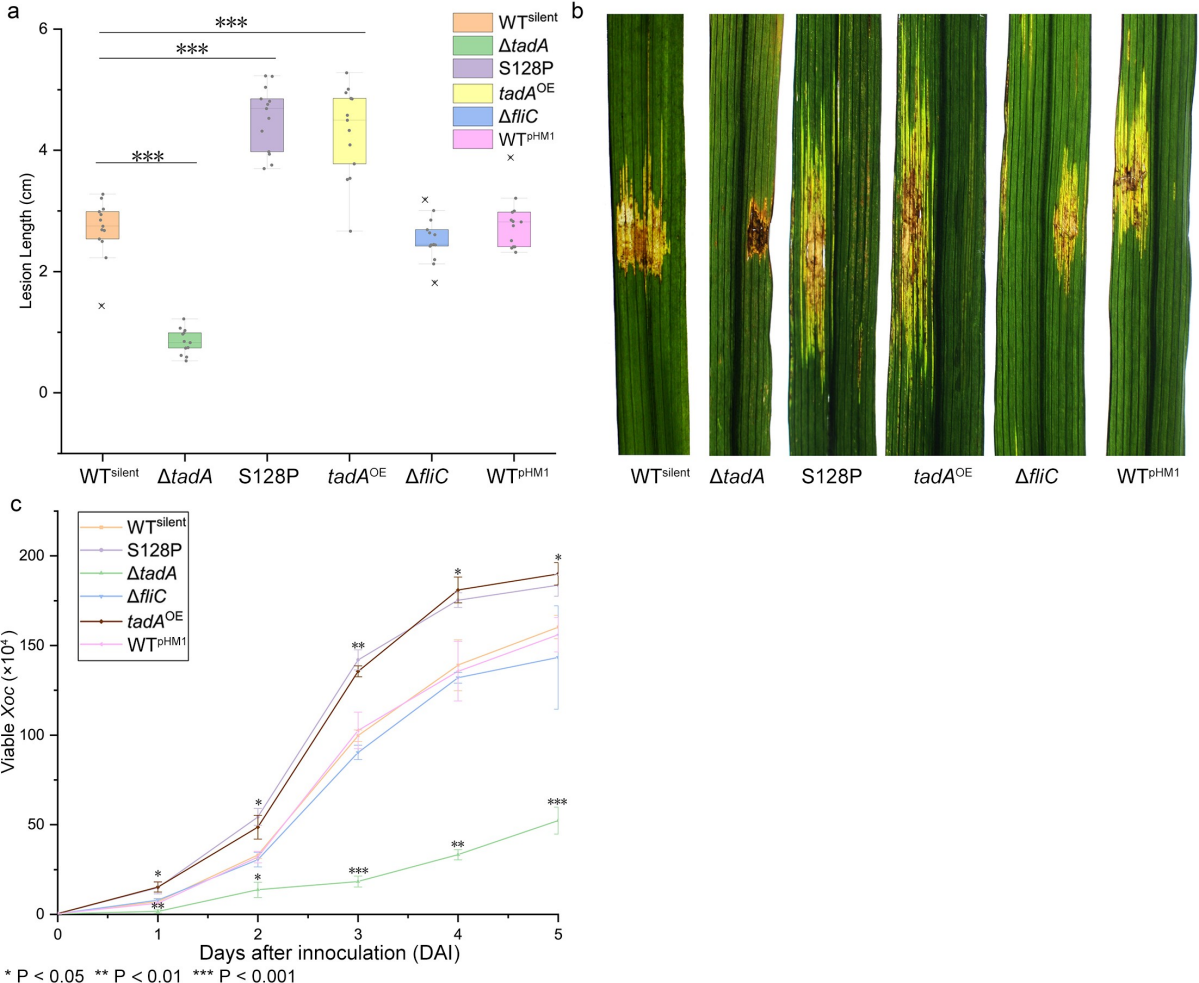
Virulence and *in planta* growth in rice cv. Zhenshan. Virulence was assessed by inoculating six-week-old susceptible Zhenshan 97 rice plants with *Xoc* strains. (a) Leaves (*n* = 12) were inoculated with needleless syringes and evaluated for lesion length 14 days after inoculation. Values represent mean lesion length ±  SD. The asterisks (***) indicate significant differences between the data obtained for Δ*tadA*, S128P and *tadA*^OE^ as compared to the WT^silent^ strain (*P* < 0.001; ANOVA with Dunnett’s multiple test post-hoc correction compared with WT^silent^). (b) Symptoms on rice leaves inoculated with the WT^silent^, S128P mutant, Δ*tadA*, *tadA*^OE^ and WT^pHM1^. (c) Population dynamics of WT^silent^, S128P, Δ*tadA*, *tadA*^OE^ and WT^pHM1^ cells *in planta* (means ± SD). Three leaves, each infected with one strain of *Xoc*, were excised around the inoculation site and then crushed with sterile beads. Each suspension was subjected to a 10-fold serial dilution, and 5 μl drops from the 10^−1^–10^−6^ dilutions were placed on NB agar with cephalexin. *, *P* < 0.05; **, *P* < 0.01, ***, *P* < 0.001.

Leaves of six-week-old cv. Zhenshan 97 (Fig 5) and four-week-old Yuanfengzao (S6 Fig) were inoculated with *Xoc* strains and mutants. At 14 days post-inoculation, lesions induced by the S128P mutant were 4.53 cm; this was significantly larger than the 2.73 cm lesions induced by WT^silent^ (Fig 5A). There was a clear decrease in the virulence of the Δ*tadA* mutant, while *tadA*^OE^ exhibited increased virulence (Fig 5A and S6A Fig), suggesting that RNA editing levels contribute to virulence. Furthermore, *in planta* growth assays indicated that the populations of the S128P mutant and *tadA*^*O*E^ were significantly higher than the other strains (Fig 5C and S6C Fig). In contrast, the Δ*fliC* strain was not significantly altered in virulence when compared to the WT, which is consistent with previous findings [28].

### S128P editing upregulates genes related to siderophore biosynthesis

**Fig 6:**
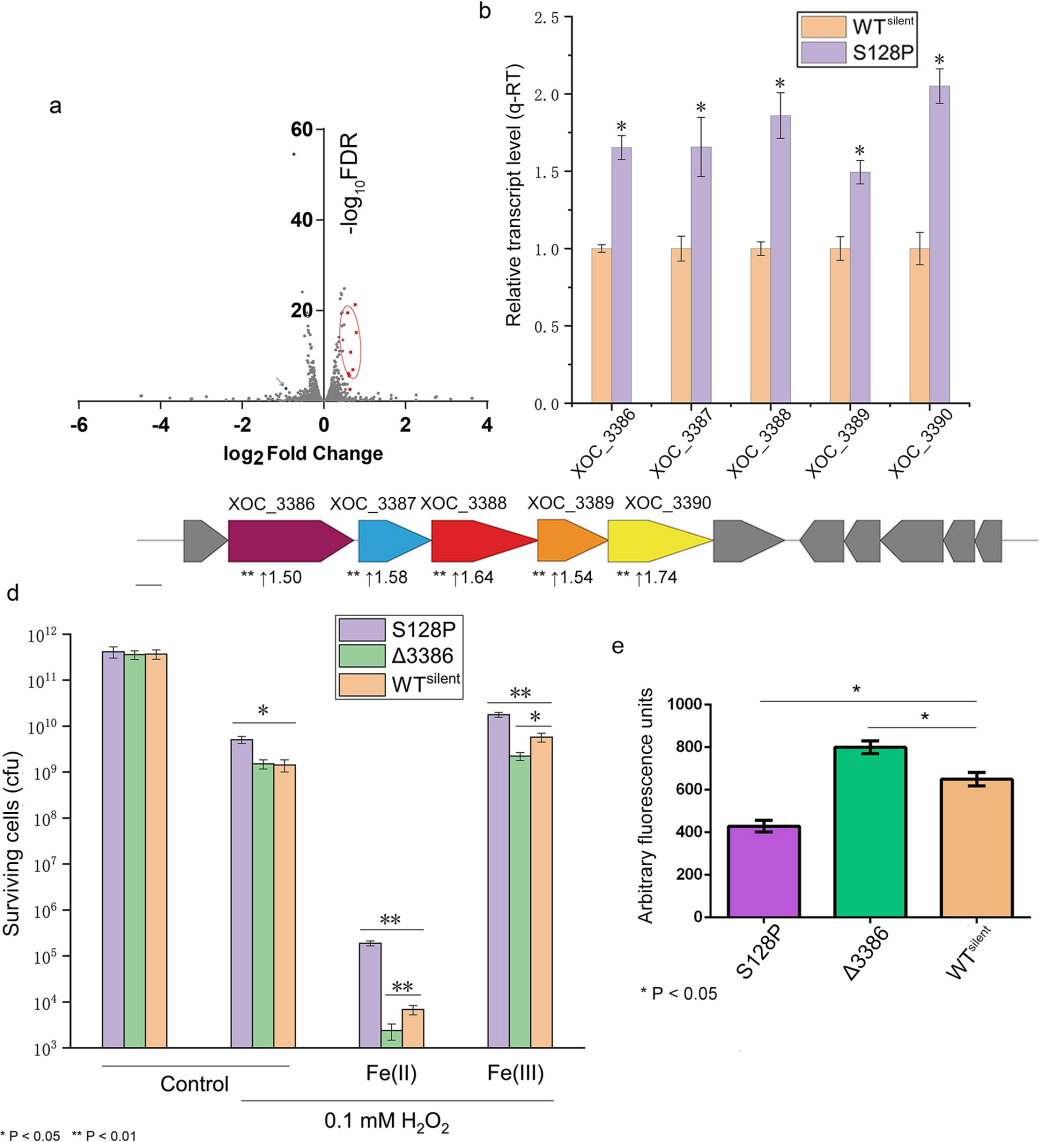
The upregulation of a siderophore biosynthesis gene cluster, which was detected by RNA-seq, contributes to a reduction in the Fenton reaction. (a) Volcano plot showing the FDR *P*-value and fold change of S128P versus WT. Red dots indicate the upregulated genes with FDR *P* < 0.01, while blue dots indicate downregulated genes with FDR *P* < 0.01. Blue arrow indicates XOC_1643, which can cause side effects to S128P as shown in S8 Fig. The circle indicates the gene cluster. (b) Normalized expression levels of target genes were calculated relative to *rpoD* using the ΔΔCT method, where CT is the threshold cycle. Four independent biological replicates were carried out in this study. (c) The organization of the gene cluster involved in siderophore biosynthesis. Vertical arrows indicate the upregulated genes in the RNA-seq data. (d) The indicated strains grown to the stationary phase were exposed to 0.1 mM H_2_O_2_ by exogenous Fe^2+^ and Fe^3+^ for 45 min and the viability of the cells was determined. Data shown are the average and SD from three independent experiments. (e) ROS accumulation by 0.1 mM H_2_O_2_ was measured based on fluorescence in microplates, the wells of which contained a bacterial suspension and no additional agent. Values are means and standard deviations from eight independent experiments. The fluorescence signal of DCF-DA converted from DCFH-DA due to oxidation. In (d) and (e), * indicates a significant difference by ANOVA with Dunnett’s multiple test post-hoc correction compared with control (WT^silent^) under the same test conditions. (*P < 0.05, **P < 0.01, ***P < 0.001).

The change in filament structure does not fully explain the increased virulence of the S128P mutant; thus, we used RNA-seq to compare the S128P and WT^silent^ strains. Before comparing RNA-seq profiles, reproducibility was evaluated for two replicate experiments using pairwise linear correlation analysis. The correlation coefficients (*r*) between the two replicate experiments were 0.980 and 0.997, suggesting satisfactory reproducibility of RNA-seq data under the experimental conditions. Based on a stringent FDR (<0.01) as a cutoff, ten differentially expressed genes were identified (S3 Table and Fig 6A). Interestingly, we identified a cluster of five up-regulated genes (*XOC_3386* to *XOC_3390*) located at positions 3478101 to 3486443 (Fig 6C). These genes were approximately 1.5-fold upregulated in S128P as compared with WT^silent^ (Fig 6B). Based on the annotations (XOC_3386, Fe^3+^ dicitrate transport protein FecA; XOC_3387, vibrioferrin biosynthesis protein PvsA; XOC_3388, vibrioferrin biosynthesis protein PvsB; XOC_3389, transport protein; XOC_3390, iron transporter), we inferred that this cluster likely contributes to siderophore biosynthesis and Fe^3+^ uptake. *XOC_3386* was annotated as *FecA*, which plays important role at iron regulation in bacteria [29]; thus, we constructed knockout mutant *Δ3386* to test the function of this gene cluster. Based on Fe^3+^ uptake Fenton reactions (Eq 1–4) [30, 31], we hypothesized that increased Fe^3+^ uptake may reduce the cell damage that is caused by H_2_O_2_, since OH· was verified as the major ROS involved in the H_2_O_2_-mediated killing of *Xanthomonas* [32].

$${Fe}^{2+}+H_{2}O_{2}\to {Fe}^{3+}+{\left(OH\right)}^{-}+OH\cdot$$ (Eq 1)

$$O_{2}\cdot^{-}+{Fe}^{3+}\to O_{2}+{Fe}^{2+}$$ (Eq 2)

$${Fe}^{3+}+HO_{2}\cdot \to O_{2}+{Fe}^{2+}+H^{+}$$ (Eq 3)

$${Fe}^{2+}+HO_{2}\cdot \to {Fe}^{3+}+HO_{2}^{-}$$ (Eq 4)

In the treatment where 0.1 mM H_2_O_2_ and 2.5 mM Fe^2+^ were added to *Xoc* cells, a precipitate appeared within 1 min indicating that a strong Fenton reaction was underway; however, this was absent when cells were suspended in H_2_O_2_ and Fe^3+^ (S7 Fig). The number of viable S128P cells was significantly higher than WT^silent^, while the number of viable *Δ3386* cells was significantly lower than WT^silent^ (Fig 6D). When cells were exposed to 2.5 mM Fe^3+^ and 0.1 mM H_2_O_2_, exogenous Fe^3+^ helped *Xoc* resist oxidative stress, but the number of the viable S128P cells was still significantly higher than the WT^silent^ strain. *Xoc Δ3386* was more sensitive to oxidative stress than WT^silent^ (Fig 6D). These results suggested that S128P can help protect cells from ROS by increasing Fe^3+^ uptake.

We also compared intracellular ROS in the S128P, WT^silent^ and *Δ3386* mutants. Compared with WT^silent^, fluorescence was significantly higher in *Δ3386* and lower in S128P (Fig 6E), suggesting that decreased uptake of Fe^3+^ could induce the ROS accumulation in *Xoc* and *vice versa*.

## Discussion

The importance of A-to-I editing in eukaryotes is well-established, but has only recently been documented in bacterial mRNA [18]. A variety of tools are available to detect edited sites in eukaryotes and use mathematic models and known SNP databases to reduce alignment errors and filter the somatic mutations [33–35]. However, these tools are not applicable to bacteria, which have smaller and less complex genomes. In this study, we developed an open source tool called AIMAP (https://github.com/castualwang/aimap), which integrates read quality control, adaptor removal, reference genome mapping, coverage pileup and synonymous-nonsynonymous annotations. AIMAP accepts Fastq-formatted files, reference genome FASTA-formatted and annotation GFF3-formatted files as minimum required inputs. Users can customize parameters by providing command line options (see pipeline website for more details). The dependencies of this pipeline include FastQC (http://www.bioinformatics.babraham.ac.uk/projects/fastqc/), Cutadapt [36], Trim Galore! (https://www.bioinformatics.babraham.ac.uk/projects/trim_galore/), BWA [37] and SAMtools [38]. This pipeline is computationally fast (80 min for a single RNA-seq sample) and reveals A to I editing sites in bacteria that have reference genomes. We predict that AIMAP will be widely used as a powerful tool in bacterial RNA editing research.

In this study, a genome-wide analysis was performed in *Xoc* with the goal of characterizing the TadA-interacting transcriptome. We identified 16 tRNAs and 30 enriched mRNA targets; most peaks mapped to tRNAs (Fig 1C), which is expected since TadA functions as a tRNA-specific adenosine deaminase [19]. The enrichment motif from RNA-seq (Fig 1B) was identical to previous report [18], which supports the accuracy of our results. However, the motif in *fliC* is GACG, which is slightly different from the enriched TACG motif. Bar-Yaacov et al. previously reported several A-to-I editing sites; 75% were embedded in the canonical motif TACG, while the rest deviated by one nucleotide (e.g. AACG, CACG, GACG and TAAG) after overexpression of *tad*A [18]. In our study, we found that a motif similar to TACG will also arises when the cells were under oxidative stress. This result may indicate that the consensus motif TACG is not as conserved when cells encounter certain external stressors. Interestingly, *fliC* and genes involved in the Type II and IV secretion systems were enriched in the iRIP-seq analysis. This suggests that the bacterial secretion system may be a target of the RNA editing machinery, and that A-to-I editing plays an important role in the adaptation of bacteria to changing conditions.

*Xoc* is a seed-borne pathogen that is continually exposed to suboptimal conditions including oxidative stress, which can damage both nucleic acids and proteins [39]. Exposure to reactive oxygen occurs both in the natural environment and also inside the plant host since ROS generation is a fundamental plant defense reaction [40, 41]. Bacteria use two main strategies to overcome oxidative stress: ROS tolerance enzymes (cytoplasmic catalase, periplasmic catalase and superoxide dismutase); and ROS scavenging, which often activates EPS production and biofilm formation [42]. Flagella are required for formation of mature biofilms in both plant and animal pathogens [21, 43]. In many bacteria, *fliC* is essential for flagellum formation; the FliC protein has a flagellin D1 domain at the N- terminus that is required for polymerization of flagellar filaments [44]. Interestingly, the Ser-to-Pro change is located in the flagellin D1 domain, which is conserved among bacteria [45]. The D1 domain of *fliC* was shown to be essential for bacterial motility, whereas deleting the other flagellin domains had no effect on motility [22]. Additionally, the S128P mutation is located at the beginning of an α-helix. Based on the Ramachandran plot, the α structures of proline are rarely observed; this is because the α-N on the side chain can form only one H-bond, which influences flagellar stability. Previous studies indicated that methylation also plays an important role in the bacterial stress resistance and flagellar performance [46–48]. For example, a recent study showed that methylation at the surface-exposed lysine residues of FliC and FljB facilitated adhesion to host cells and thus contributed to colonization and successful infection of the host [49]. These results suggest that epigenetics may play a pivotal role in bacterial stress resistance and pathogenicity.

In *Xoc* BL256, the S128P editing event occurs in about 20% of bacterial cells exposed to oxidative stress. To increase the magnitude of editing, we generated the S128P mutant to mimic a 100% editing event. This approach was undertaken in previous A-to-I editing reports [15, 18]. In most experiments, we compared the S128P mutant to the WT^silent^ mutant, which cannot be edited at the GACG motif. This allowed us to compare a strain that had 100% editing with an isogenic strain that had 0% editing.

The acquisition of iron by siderophores promotes the invasion of pathogenic microorganisms and facilitates successful infection of plant hosts [50, 51]. In this study, we identified a siderophore biosynthesis gene cluster that was upregulated in the S128P mutant. We also confirmed that upregulation was due to the A-to-I editing event rather than an off-target mutation in *fliC* by sequencing the up- and downstream regions and resequencing the coding region. The upregulation of the siderophore gene cluster due to the mutation in *fliC* was unexpected. However, previous studies have suggested that many genes can be indirectly induced or altered due to changes in flagellin [49, 52]. Subsequent experiments showed that upregulation of this cluster (*XOC_3386*-*XOC_3390*) was correlated with a decrease in the Fenton reaction, which was followed by increased biofilm formation and pathogen virulence. Biofilm formation in bacteria is associated with increased adhesion to surfaces and improved stress resistance [53, 54]; thus, we speculated that enhanced biofilm formation by the S128P mutant results in increased tolerance to oxidative stress and improved colonization of plant hosts. Interestingly, the *Xoc* effector protein AvrBs2 was predicted to participate in the siderophore protein network using the STRING database [55] and Cytoscape (S8 Fig). This is noteworthy since AvrBs2 is a known inhibitor of rice defense responses [56] in response to *Xoc*. Further studies are needed to investigate and confirm this possible interaction.

It is well-established that a multitude of factors contribute to fitness in bacteria. The trade-off hypothesis in pathogen evolution states that a higher benefit in one aspect is correlated with a reduced benefit in another aspect [57]. Therefore, in addition to the benefits of increased virulence and enhanced stress resistance, we speculate that there must be undesirable side effects derived from the S128P mutation. Interestingly, expression of *XOC_1643* (encoding aβ-lactam efflux pump protein) was downregulated in RNA-seq result, which decreases resistance toβ-lactam antibiotics (S9 Fig); this may be an example of how the S128P mutation decreases fitness, thus supporting the trade-off theory.

We show that *tadA* participates in posttranscriptional adenosine deamination, which is consistent with recent findings [18]. Interestingly, *tadA* was shown to be essential in *E*. *coli* [19] but was dispensable in *Xoc* (this study). To determine if *tadA* is essential in other bacteria, we used the OGEE tool (Online GEne Essentiality) [58]. Interestingly, *tadA* was nonessential in *Bacillus subtilis* subsp. *subtilis* str. 168 (BSU00180) and *P*. *aeruginosa* PAO1 (PA4302). We identified two nonsynonymous editing events in *Xoc* mRNA (S128P in *fliC* and T408A in *XOC_3486*). In this study, we show that the editing in the S128P mutant is controlled by *tadA*; however, the T408A editing event in *XOC_3486* was not dependent on *tadA* (S11 Fig). We speculate that other enzymes may exist that compensate for the absence of a functional TadA, and this concept warrants further study.

**Fig 7:**
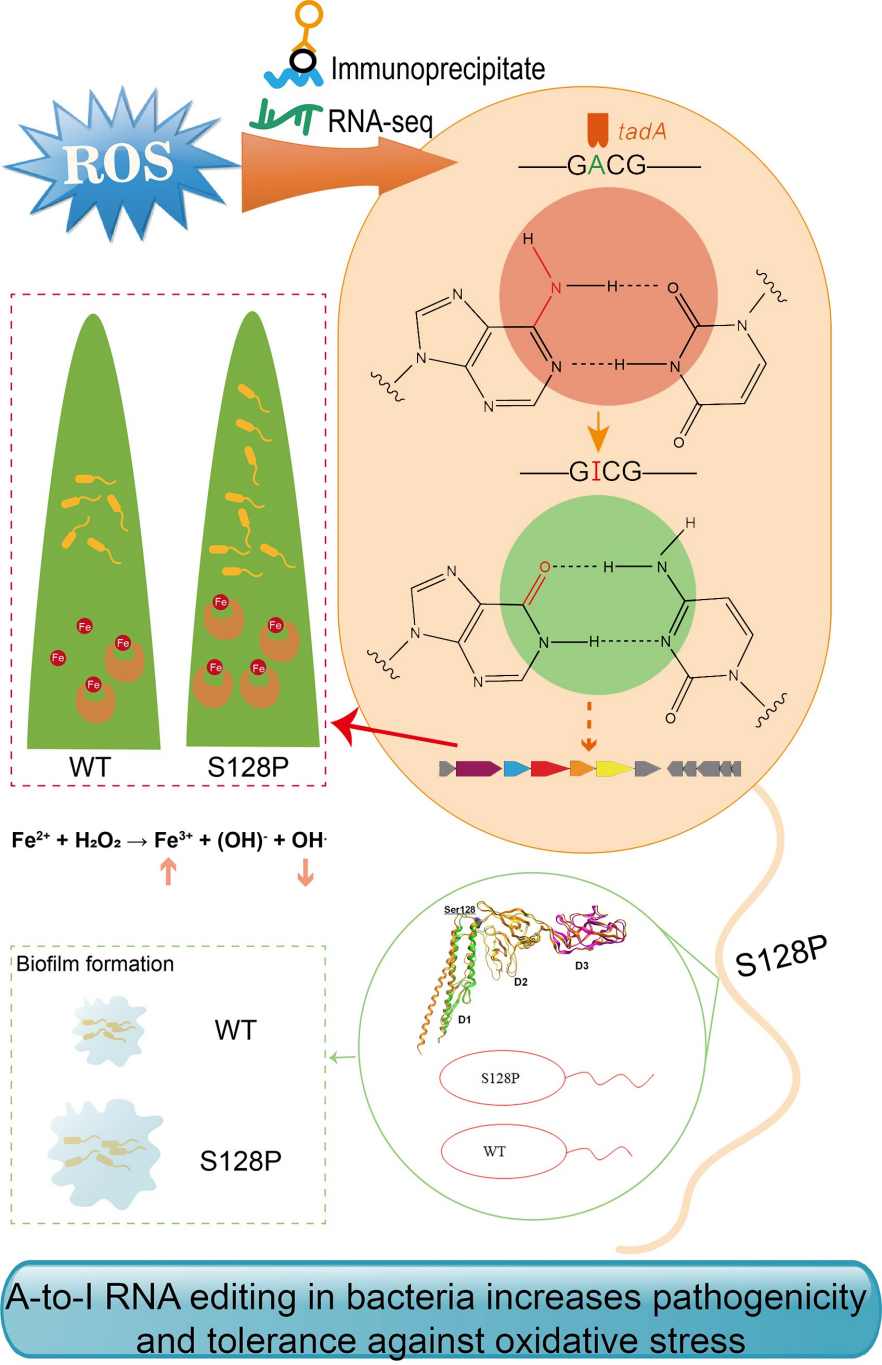
Proposed new mechanism that bacteria use to adapt to oxidative stress. When bacteria are exposed to ROS, TadA-mediated A-to-I editing occurs in *fliC* mRNA at the GACG motif. The S128P editing event within FliC can enhance tolerance to oxidative stress, potentially due to the change in filament structure and enhanced biofilm formation. RNA-seq revealed a siderophore biosynthetic gene cluster that was upregulated in S128P compared with WT; the enhanced transcription of this cluster could contribute to a decrease in the Fenton reaction and increased biofilm formation and virulence.

In summary, our study describes a new mechanism for bacterial adaptation to oxidative stress (Fig 7). When bacteria are exposed to ROS, TadA-mediated A-to-I editing occurs in *fliC* mRNA at the GACG motif. The editing event within *fliC* increases tolerance to oxidative stress, potentially due to the change in filament structure and enhanced biofilm formation. Using RNA-seq, a siderophore biosynthetic gene cluster was shown to be upregulated in the S128P mutant, which could explain oxidative stress tolerance and increased biofilm formation (Fig 7). Overall, our work demonstrates that A-to-I RNA editing is a novel mechanism that pathogens use to adapt to ROS and promote virulence.

## Materials and methods

### Bacterial strains, plasmids, and plant materials

The bacterial strains and plasmids used in this study are described in S5 Table. *Xoc* BLS256 is the wild-type (WT) strain of *X*. *oryzae* pv. *oryzicola*. WT^silent^ contains a synonymous mutation in *fliC* that blocks A-to-I RNA editing at amino acid 128. Strain S128P is a derivative of BLS256 containing a mutation that changed the serine residue at amino acid 128 to proline. Strain *tadA*^OE^ is a derivative of BLS256 that overexpresses *tadA* in vector pHM1; WT^pHM1^ is BLS256 harboring the pHM1 empty vector. Further details on strains and plasmids are provided in S5 Table.

*Escherichia coli* strains were cultured in Luria-Bertani (LB) medium at 37°C [59]. *Xoc* BLS256 and *P*. *putida* KT2440 strains were cultured in nutrient broth (NB, 1% peptone, 0.5% yeast extract, 1% sucrose) or in NB containing 1.5% (w/v) agar at 28°C. The final concentrations of the antibiotics were as follows: ampicillin, 100 μg/mL; cephalexin, 40 μg/mL; kanamycin, 25 μg/mL; and spectinomycin, 50 μg/mL.

Rice seeds were obtained from the International Rice Research Institute. All rice plants were grown at 28°C with a 12-h photoperiod in a greenhouse located at Shanghai Jiao Tong University.

### Bacterial mutant and strain construction

Construction of bacterial mutant strains was performed as described by Baba [60] with the following modifications. Two fragments flanking *tadA* in *Xoc* BLS256 were amplified with *Pfu* polymerase (TransGen Biotech, Beijing, China) using the *tadA* up- and downstream primers (S6 Table). These fragments were cloned, digested with *Pst*I/*Sma*I and *Sma*I/*Xba*I, and the two fragments were ligated into the *Pst*I/*Xba*I site of suicide vector pKSM1 [61]. Similarly, fragments flanking *fliC* in BLS256 were amplified with *fliC* up- and downstream primers (S6 Table), digested with *Bam*HI/*Sma*I and *Sma*I/*Xba*I, and subcloned into the *Bam*HI/*Xba*I site of pKSM1. Fragments flanking *XOC_3386* were amplified using the up- and downstream fragments in S6 Table, digested with *Pst*I/*Eco*RI and *Eco*RI/*Xba*I, and subcloned into the A/B site of pKSM1.

To construct the Δ*tadA*, Δ*fliC*, and Δ*3386* deletions, the fragments flanking *tadA*, *fliC*, and *XOC_3386* in pKMS1 were separately introduced into *Xoc* BLS256 by electroporation (Bio-Rad Laboratories Inc., Hercules, CA, USA) and subjected to kanamycin selection. A single transformant colony with kanamycin resistance was picked and cultured for 8 h in NB medium without kanamycin and sucrose; 10-fold dilutions were then inoculated to nutrient broth agar (NA) with 15% sucrose to obtain sucrose-insensitive clones.

For site-directed mutagenesis, plasmids were modified with the Fast Mutagenesis System (Transgen Biotech, Beijing, China) to obtain clones containing the point mutations (S128P and WT^silent^). Fragments encoding *fliC* in *Xoc* BLS256 and *P*. *putida* KT2440 were amplified with corresponding *fliC* F/R primers (S6 Table), digested with *Bam*HI/*Xba*I, and subcloned into the *Bam*HI/*Xba*I site of pKMS1. Mutations were introduced by PCR amplification with overlapping primers containing the target A-G point mutations as follows: S128P F/R; *Xoc*^silent^ F/R; and S491P F/R (S6 Table). The PCR product was digested with the DMT enzyme supplied in the Fast Mutagenesis System, and plasmids containing A-G point mutations were transformed into DMT competent cells and verified by Sanger sequencing. Sequence substitutions were performed by double homologous recombination similar to the gene knockouts described above.

To obtain a derivative of BLS256 overexpressing *tadA* (designated *tadA*^*OE*^), full-length *tadA* was amplified with the *tadA* F/R primers (S6 Table) and cloned as a *Sal*I/*Hin*dIII fragment in pHM1. The recombinant plasmid was transferred into BL256 by electroporation, and transformants were screened on NA plates supplemented with spectinomycin.

To construct the strain used for iRIP-Seq, a 6X-His-tag was introduced by overlapping PCR prior to the stop codon of *tadA* in *Xoc* BLS256 (His-BLS256, S5 Table). Briefly, fragments flanking the stop codon *tadA* in BLS256 were amplified with *tadA*HT up- and downstream primers (S6 Table), digested with *Pst*I/*Xba*I and subcloned into the *Pst*I/*Xba*I site of pKSM1. The 6X-His-tag sequence was introduced by two overlapping primers, *tadA*Ht upstream R and *tadA*Ht downstream F (S6 Table). The plasmid was verified by Sanger sequencing and the His-Tag was introduced via double cross-over homologous recombination.

pHM1::*gfp* (S5 Table) was introduced into S128P, Δ*fliC*, and WT^silent^ by electroporation, Sp^R^ colonies were selected, and strains were verified by confocal microscopy.

The S491P mutation was constructed by introducing the S491P::pKMS1 plasmid into *P*. *putida* KT2440 by electroporation with kanamycin selection. A single Km^R^ transformant was selected and cultured for 8 h in LB medium without kanamycin and with 15% sucrose; 10-fold dilutions were then inoculated to LB agar with 15% sucrose to obtain sucrose-insensitive clones.

### Development of the A-to-I modification analysis pipeline

We developed an open source tool called AIMAP (A-to-I modification analysis pipeline), which is implemented in Python 3 and runs in the Linux OS. It is freely available at https://github.com/castualwang/aimap under the MIT license with external binaries bundled in the software. S1 Fig shows a schematic depiction of the AIMAP pipeline. In the default configuration, the pipeline processed data in the following order:

### 1. Read quality control

FastQC (http://www.bioinformatics.babraham.ac.uk/projects/fastqc/) was implemented along with Cutadapt [36] and Trim Galore! (https://www.bioinformatics.babraham.ac.uk/projects/trim_galore/), which were used to trim off adaptor sequences and low quality base calls from the end of sequencing reads. This process is important for the identification of modified sites.

### 2. Read alignment and pileup

Data was aligned using BWA [37] and default parameters. SAMtools [38] was used to pileup the aligned sequences, and the coverage of every base on the chromosome was calculated.

### 3. A-to-I modification detection and annotation

To increase accuracy, modified sites had to be supported by at least one mismatched read with minimum coverage ≥30; 3% frequency was used as default parameter.

The AIMAP output file provided information on chromosome position, reference site (base position), modified sites, total coverage, % base change, coding region, gene name/product and whether the annotation was synonymous or nonsynonymous.

### Analysis of A-I mutations

The frequency of editing was estimated by ratiometric A/G measurement from Sanger sequencing [9]. The effect of mutations with amino acid substitutions was predicted using Protein Variation Effect Analyzer (PROVEAN) (http://provean.jcvi.org/genome_submit_2.php). This tool provides PROVEAN and Sorting Intolerant from Tolerant (SIFT) predictions for each mutation.

PCR (Edit F/R primers, S6 Table) was performed to sequence the *fliC* gene/transcript from DNA/RNA samples of *Xoc* BLS256, Δ*tadA* and *tadA*^OE^. Chromatograms were visualized by Chromas Lite (Technelysium, Brisbane, Australia), and the frequency of editing was estimated by ratiometric A/G measurement.

### RNA immunoprecipitation–coupled high-throughput sequencing (iRIP-Seq)

iRIP-Seq was conducted as described previously with minor modifications [62]. His-BLS256 cells (10^9^) were cross-linked on ice with UV irradiation at 400 mJ/cm^2^, harvested by centrifugation (1,000 *g* at 4°C), and lysed for 10 min in ice-cold wash buffer [62] containing RNase inhibitor (Takara, Beijing, China) and protease inhibitor cocktail (Roche, Shanghai, China). Lysates were centrifuged at 16,000 *g* for 20 min at 4°C; RQI RNase-Free DNase (Promega Biotech, Wisconsin, USA) was added and lysates were incubated for at 37°C (5 min) and then cooled on ice (5 min).

For immunoprecipitation, supernatants were incubated with 15 μg His-Tag antibody (Thermo Fisher, Waltham, USA) overnight at 4°C; immunoprecipitates were then incubated with protein A or G Dynabeads (Thermo Fisher) for 3 h at 4°C. After removal of the supernatants with a magnet, beads were washed with lysis buffer, high-salt wash buffer, and polynucleotide kinase (PNK) buffer as described [62]. After addition of 1:1,000,000 MNase (300 U/μL; Fermentas, Waltham, Massachusetts, USA), beads were resuspended, incubated for 15 min at 37°C, and supernatants were removed. The beads were washed 2X in PNK buffer and dephosphorylated with calf intestinal phosphatase (New England Biolabs, Massachusetts, USA) for 10 min at 37°C. The beads were then washed 2X in PNK buffer without DTT and then resuspended in PNK buffer containing ATP and T4 PNK (New England Biolabs) as described [62]. The suspension was incubated at 37°C, washed in PNK buffer (without DTT), resuspended in 150 μL SDS+DTT elution buffer, and incubated at 70°C for 20 min to denature and release cross-linked RNAs [62]. Beads were removed, and the supernatant was transferred to a sterile microfuge tube and treated with proteinase K (Roche) as described [62].

RNAs were recovered by acidic phenol/chloroform/isoamyl alcohol extraction, followed by chloroform extraction; glycogen was added and RNAs were precipitated as described [62]. Recovered RNAs were used to generate a paired-end sequencing library with the TruSeq Small RNA Library Preparation kit (Illumina) as recommended by the manufacturer. Libraries were purified, quantified, and stored at -80°C until sequencing, which was conducted with the Illumina NextSeq 500 system using 150-nt paired-end sequencing.

### Bacterial growth assays in response to oxidative stress

Optical density (OD) was measured with a Bioscreen C instrument (Labsystem, Helsinki, Finland). For each well, 99 μL of NB or LB broth with or without H_2_O_2_ was inoculated with 1 μL of an overnight culture (1×10^9^ CFU/mL). The OD values at 420–580 nm were measured every 15 min for 48 h with continuous shaking at 28°C (*Xoc*) or 30°C (*P*. *putida* KT2440).

For viable plate counts, overnight suspensions (1×10^9^ CFU/mL) were diluted 100-fold with fresh NB with or without 0.1 mM H_2_O_2._ At 4 h intervals, 100 μL aliquots were diluted (10-fold, 10 times), and 100 μL of diluent was inoculated to NA. After incubation at 28°C for 36 h, colony numbers were determined. All experiments were performed in quadruplicate, and the Kolmogorov–Smirnov test was used to evaluate significance.

### 3D structure prediction

For 3D modeling, the sequence of the WT FliC was input into the RCSB Protein Data Bank (PDB) to search for templates that could be used for homology modeling. The X-ray structure of *P*. *aeruginosa* flagellin 4NX9 was closely related with *Xoc* FliC, with a sequence identity score of 58%. Based on the known structure of 4NX9, residues 81–369 of FliC were subjected to homology modeling using SAMM [63]. This partial model was locally mutated at residue Ser128 to proline. Both the WT FliC and Ser128Pro FliC mutant were subjected to energy minimization using a CHARMM22 force field [64]. Default values were used for the iteration limit and RMS gradient test.

### Prediction of RNA secondary structure

A sequence consisting of residues from -25 to +25 bp from the S128P edited site was extracted for prediction of RNA secondary structure using the RNAfold web server (http://rna.tbi.univie.ac.at/cgi-bin/RNAWebSuite/RNAfold.cgi) based on minimum free energy.

### Measurement of flagellar length and radius

Transmission electron microscopy (TEM) was used to observe bacterial morphology and measure filament length and radius. Bacteria were prepared by inoculating 1 μL of an overnight suspension onto NB agar. Plates were incubated at 28°C for 24 h, washed with 10 mM PBS (pH = 7.4), fixed with 2.5% (w/v) glutaraldehyde in PBS and collected on copper grids. Samples were then dehydrated and observed by TEM (HT-7700, Hitachi, Japan). Forty cells of each sample were analyzed from each of three biological replicates, giving sample sizes of 120 cells for each data set. Filament length and width was measured to the nearest 0.01 μm. ImageJ software was used to process images and calculate filament length and radius [65].

### Swimming motility assay

Clean coverslips were attached to slides with double-sided tape to create a chamber, and bacterial suspension (40 μL) was added to the chamber to measure swimming motility. After sample preparation, videos of swimming bacteria were immediately recorded using phase contrast microscopy (Nikon Ti-E). Videos were obtained by capturing 2000 frames near the coverslip surface with a Thorlabs CMOS camera (DCC1240M) at a framerate of 25 fps at 40X magnification.

The videos were analyzed with self-coded MATLAB script. To obtain the speed distributions of each strain, all instantaneous velocities were taken into account. To eliminate crippled data, only tracks with tracking time longer than 2 s were calculated, and a mean speed threshold was used according to the mean velocity distribution. Mean values and standard deviation of speed were calculated from fitting the second peak in the speed distribution with Gaussian function. The propulsion force was calculated as described previously [66]. Briefly, the equation used was as follows:

$$F_{p}=\left(\frac{4\pi \eta l}{ln\frac{2l}{r}-\frac{1}{2}}\right)\times v$$

where *v* is the swimming velocity of the bacteria; *η* is the viscosity of the swimming buffer; *l* and *r* represent length and radius of the flagellum.

### Visualization of bacterial biofilms

Due the production of extracellular polymers, the traditional fluorochrome method is unsuitable for visualization of biofilm formation by *Xoc*; hence we constructed GFP-labeled strains to evaluate biofilm formation using a protocol similar to that described by Gowrishankar et al. [67]. Briefly, microscope slides were sterilized and placed on the bottom of aseptic petri dishes containing 10^9^ CFU of GFP-labeled bacteria in 1 mL NB medium. The petri dishes were incubated at 28°C for 96 h and then gently washed five times with 1 mL sterile water to remove non-adherent cells. The default GFP setting of the confocal laser scanning microscope (CLSM, Leica TCS SP5, Leica, Germany) was selected with a resolution of 1024×1024 at 40X magnification. CLSM images (*n* = 20) were randomly obtained from 12 replicates, and the volume of Z-stack was 10.20 μm at 0.34 μm per Z-step. The images, surface topographies and 3D architecture were processed with Leica Application Suite X version 3.4.2.18368).

For SEM, samples were prepared as described by Asahi [68] with minor modifications. Briefly, biofilm formation was initiated on sterile glass coverslips that were incubated at 28°C for 96 h. A hydrophilic ionic liquid (10% [C_2_mim][AcO]), was used to steep the biofilm samples for 10 min at 25°C, excess liquid was removed, and specimens were observed with field-emission SEM (Sirion-200 SEM, JEOL, Japan) using the secondary electron emission mode at 5,000 V. Specimens were viewed at 1,500X and 5,000X.

### Plant inoculation assays

Bacterial suspensions (OD_600_ = 0.6) were used to inoculate 6-week-old rice with needleless syringes, and lesion lengths were measured 14 days after inoculation. Twelve or more leaves were inoculated and evaluated for each *Xoc* strain.

For colony counting, three leaves, each infected with one *Xoc* strain, were excised around the inoculation site and then crushed with sterile beads. Each suspension was subjected to a 10-fold serial dilution, and 5 μL aliquots from 10^−1^ to 10^−6^ dilutions were inoculated to NA with cephalexin. Plates were incubated at 28°C until individual colonies could be counted; statistical analysis was used to calculate viable bacteria per leaf.

### mRNA purification and cDNA synthesis for RNA sequencing

Total RNA (10 μg/sample) was isolated and treated with the MICROBExpress Bacterial mRNA Enrichment kit (Ambion) and RiboMinus Transcriptome Isolation Kit (Bacteria) (Invitrogen) as recommended by the manufacturers. Bacterial mRNAs were suspended in RNase-free water (15 μL) and chemically fragmented to 200–250 bp using 1X fragmentation solution (Ambion) for 2.5 min at 94°C. cDNA was generated according to instructions provided in the SuperScript Double-Stranded cDNA Synthesis Kit (Invitrogen). Briefly, mRNA samples were mixed with 100 pmol of random hexamers, incubated at 65°C for 5 min, chilled on ice, and mixed with 4 μL of First-Strand Reaction Buffer (Invitrogen), 2 μL 0.1 M DTT, 1 μL 10 mM RNase-free NTP mix, and 1 μL SuperScript III reverse transcriptase (Invitrogen); mixtures were then incubated at 50°C for 1 h. To generate the second strand, the following Invitrogen reagents were added and samples were incubated at 16°C for 2.5 h: 51.5 μL RNase-free water, 20 μL second-strand reaction buffer, 2.5 μL 10 mM RNase-free dNTP mix, 50 U DNA polymerase, and 5 U RNase H.

### RNA sequencing and RNA-seq data analysis

The Illumina Paired End Sample Prep kit was used to create RNA-Seq libraries as described previously [20]. After removing low quality reads and adaptors, RNA-Seq reads were aligned to the corresponding *Xoc* BLS256 genome using Tophat 2.0.7 [69], allowing for a maximum of two mismatched nucleotides. If reads mapped to more than one location, only the site showing the highest score was retained. Reads mapping to rRNA and tRNA regions were removed from further analysis. After obtaining read numbers from every sample, edgeR with TMM normalization method was used to determine differentially expressed genes (DEGs). Significant DEGs (FDR value < 0.01) were selected for further analysis, and Cluster 3.0 and Treeview 1.1.6 were used to generate heatmap clusters based on the RPKM values [70, 71].

### qRT-PCR

RNA was purified with EasyPure RNA Kit (Transgen Biotech) as recommended by the manufacturer. RNA (1 μg) was used to synthesis cDNA with TransScript II One-Step gDNA Removal and cDNA Synthesis SuperMix (Transgen Biotech). Then, 20 μL of synthesized cDNA was diluted to 100 μL and used for qRT‐PCR with TransStart Tip Green qPCR SuperMix (TransGen Biotech) and the ABI 7500 quantitative PCR system (Applied Biosystems, Foster City, CA). Normalized expression levels of target genes were calculated relative to *rpoD* using the ΔΔCT method, where CT is the threshold cycle. Four independent biological replicates were carried out in this study.

### Measurement of intracellular ROS

ROS levels in S128P and WT^silent^ were measured using a reactive oxygen species assay kit (Beyotime, China) according to the manufacturer's protocol. This kit detects the ROS with the DCFH-DA (2,7-dichlorofluorescein diacetate) probe. DCFH-DA can pass through the cell membrane and would be hydrolyzed by intracellular esterase to produce DCFH (2,7-dichlorofluorescein). DCFH disrupts the membrane permeability and the intracellular ROS would oxidize DCFH to produce fluorescent DCF (dichlorofluorescein). The level of intracellular ROS was measured by detection of fluorescence via DCF. Briefly, after a 15-min exposure to 0.1 mM H_2_O_2_, 1 mL of bacterial cells (10^9^ CFU/mL) was treated with 30 μL 10 μM DCFH-DA and incubated at 28°C for 20 min. After the extracellular DCFH-DA was removed, the bacterial cells were collected by centrifugation at 4,000 *g* for 5 min, washed three times and re-suspended in fresh medium. Cells (200 μL) were analyzed using a multimode reader (ENSPIRE 2300, Promega, USA) with excitation and emission wavelengths of 488 and 525 nm, respectively. Eight biological replicates were analyzed in this experiment.

### Fe^2+^/Fe^3+^ sensitivity assays

Fe^2+^/Fe^3+^ sensitivity assays were performed as described previously with minor modifications [72]. Stationary phase *Xoc* cells were harvested by centrifugation, washed with M9 medium containing Fe^2+^ or Fe^3^, and exposed to 0.1 mM H_2_O_2_ for 40 min at 28°C. Cultures were subjected to serial 10-fold dilutions, inoculated to NB agar, and colonies were counted after a 36 h-incubation at 28°C. This assay was performed in triplicate.

### Exposure of *Xoc* strains to oxidative stress and RNA extraction

*Xoc* strains were exposed to H_2_O_2_ as described previously with minor modifications [73]. Bacteria were cultured to the midlog phase (OD_600_ = 1.0–1.2) in NB medium, and then exposed to 0.1 mM H_2_O_2_ in a 28°C shaking incubator. At 0, 7, 15, and 45 min intervals, aliquots were withdrawn and pelleted by centrifugation at 4°C. Each cell pellet was washed two times in cold PBS, and total RNA was then extracted with the RNeasy Protect Bacteria Mini Kit (Qiagen). Two biological replicates were examined for this experiment.

## Supporting information

S1 Fig Workflow of the AIMAP Pipeline. (TIF)

S2 Fig Growth of *Xoc* WT^silent^, S128P, Δ*tadA*, Δ*fliC*, *tadA*^OE^ and WT^pHM1^ in NB medium. Panels show cell counts in NB medium (a) and NB medium supplemented with 0.1 mM H_2_O_2_ (b). Data points represent mean viable cell counts from quadruplicate samples.
(TIF)

S3 Fig RNA secondary structure prediction of the S128P mutation in *Xoc* FliC. RNA secondary structure analysis (http://rna.tbi.univie.ac.at/) showed that the edited site is embedded within a loop (see arrow).
(TIF)

S4 Fig S128P editing has no effect on swimming speed. Bacterial cell velocity was randomly traced for 575 and 721 bacterial cells from S128P and WT^silent^, respectively.
(TIF)

S5 Fig SEM images of biofilms prepared for observation using hydrophilic 10% [C_2_mim][AcO]. Panels: (a) WT^silent^; (b) S128P mutant; and (c) Δ *fliC* mutant.
(TIF)

S6 Fig Virulence and *in planta* growth of *Xoc* strains in rice cv. Yuanfengzao. Virulence was assessed by inoculating six-week-old susceptible rice plants. (a) Twelve leaves were inoculated per strain with needleless syringes, and lesion lengths were evaluated 14 days after inoculation. Results indicate means ±  SD; asterisks (***) show significant differences between mutants and WT^silent^ (*P* < 0.001; ANOVA with Dunnett’s multiple test post-hoc correction compared with WT^silent^). (b) Symptoms on rice leaves inoculated with *Xoc* WT^silent^, S128P mutant, Δ*tadA*, *tadA*^OE^ and WT^pHM1^. (c) Number of viable WT^silent^, S128P, Δ *tadA*, *tadA*^OE^ and WT^pHM1^ cells *in planta*. Population dynamics of bacterial strains in infected leaves (means ± SD). Infiltrated regions were examined in three independent experiments (three leaves each from three independent experiments) for each strain of *Xoc*, *, *P* < 0.01; **, *P* < 0.001.
(TIF)

S7 Fig *Xoc* WT cells washed with M9 medium containing 0.1 mM H_2_O_2_ containing exogenous Fe^2+^ or Fe^3+^. Precipitates were products of the Fenton reaction.
(TIF)

S8 Fig Predicted interaction network for the siderophore biosynthetic cluster and other genes investigated in this study; confidence hits are based on String analysis. Line thickness correlates with the evidence supporting the interactions among proteins.
(TIF)

S9 Fig Comparison of the *Xoc* WT^silent^ and S128P mutant strain growth in NB medium supplemented with ampicillin, 250 μg/mL. (TIF)

S10 Fig Comparison of *P*. *putida* KT2440 WT and the S491P mutant in NB medium. The strains were grown in quadruplicate to mid-exponential phase in NB, diluted to OD_600_ = 0.1, transferred to fresh NB and placed in a Bioscreen C apparatus at 28°C to monitor growth. Panels: (a) growth of *P*. *putida* KT2440 and S491P mutant in NB; (b) growth in NB supplemented with 2 mM H_2_O_2_; and (c) growth in NB supplemented with 4 mM H_2_O_2_. Data points represent mean OD values.
(TIF)

S11 Fig RNA-seq and Sanger sequencing results from T408A editing event in *XOC_3486*: (a) editing level from RNA-seq; (b) editing level from Sanger sequencing.
(TIF)

S1 Video Swimming motility in *Xoc* WT^silent^ and the S128P mutant. Videos were recorded using phase contrast microscopy.
(MP4)

S2 Video 3D movie of biofilms produced by *Xoc* WT^silent^, the S128P mutant, and the Δ*fliC* mutant using confocal laser scanning microscopy. (MP4)

S1 Table AIMAP output based on the raw data from Bar-Yaacov, et al., 2017. (XLSX)

S2 Table A to I mRNA editing in Xoc after exposure to H_2_O2. (XLSX)

S3 Table Differentially expressed genes in Xoc WT^silent^ and S128P mutant. (XLSX)

S4 Table Potential A to I editing sites in *P*. *putida* KT2440 exposure to H_2_O2. (XLSX)

S5 Table Strains and plasmids used in this study. (DOCX)

S6 Table Primers used in this study. (DOCX)
