# Supplementary figures and images for: A-to-I RNA editing in bacteria increases pathogenicity and tolerance to oxidative stress
Source: PLoS Pathog. 2020 Aug 21;16(8):e1008740. doi: 10.1371/journal.ppat.1008740 (PMC7467310; doi:10.1371/journal.ppat.1008740)

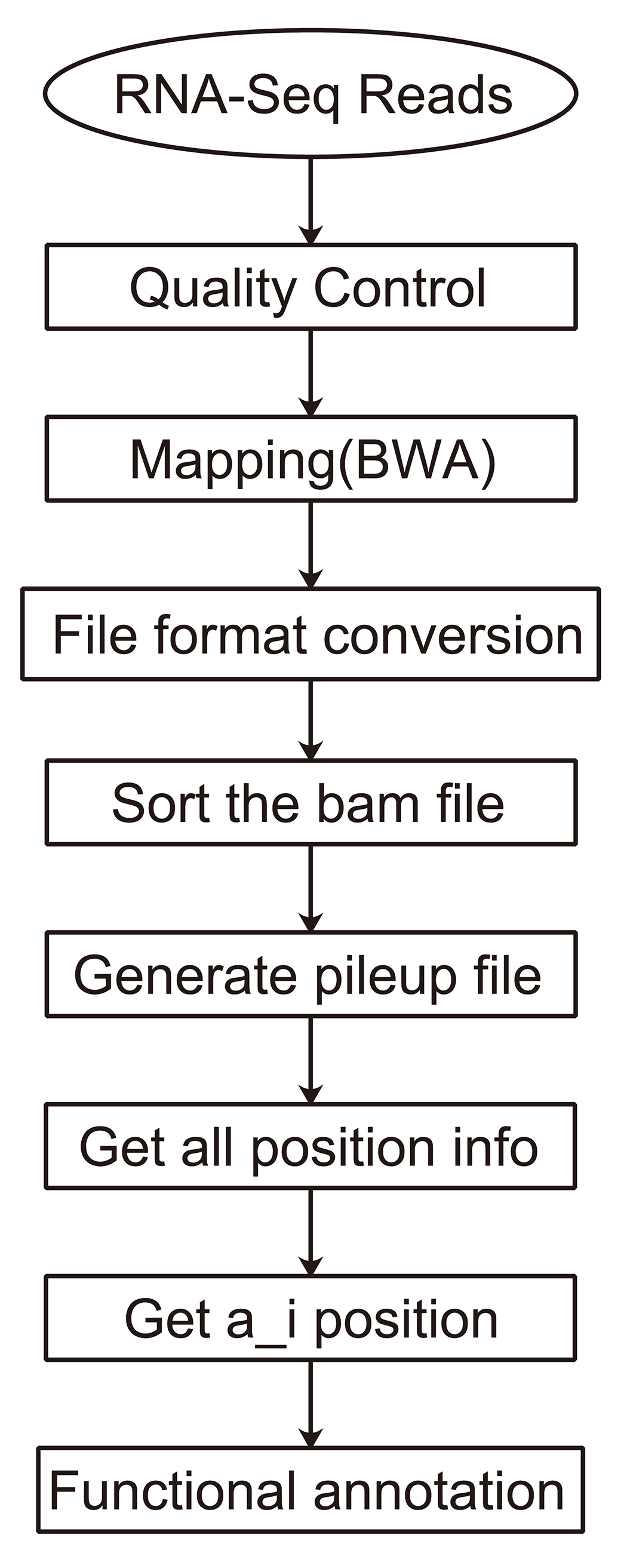

Supplement: S1 Fig — (TIF) [file ppat.1008740.s001.tif]

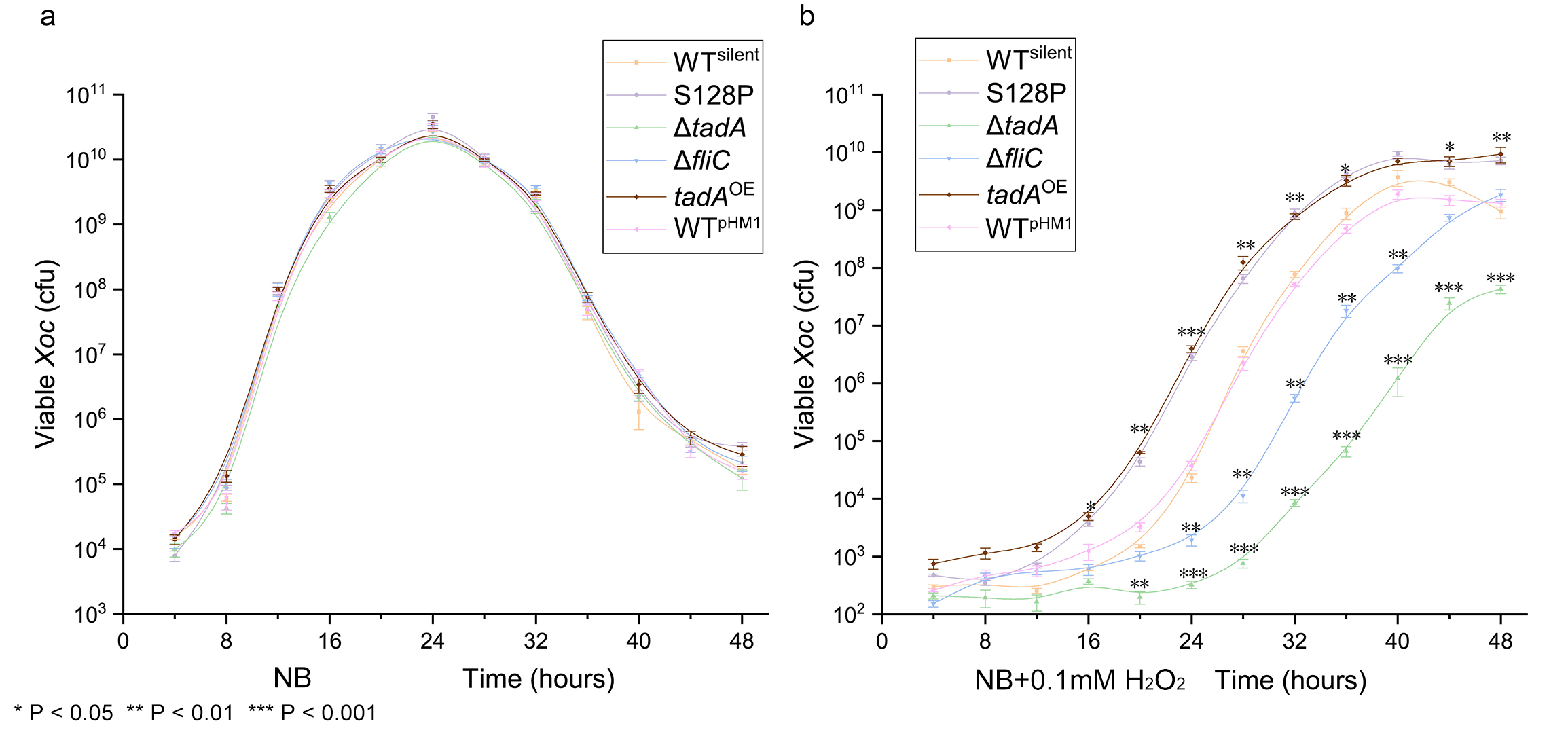

Supplement: S2 Fig — Panels show cell counts in NB medium (a) and NB medium supplemented with 0.1 mM H2O2 (b). Data points represent mean viable cell counts from quadruplicate samples. (TIF) [file ppat.1008740.s002.tif]

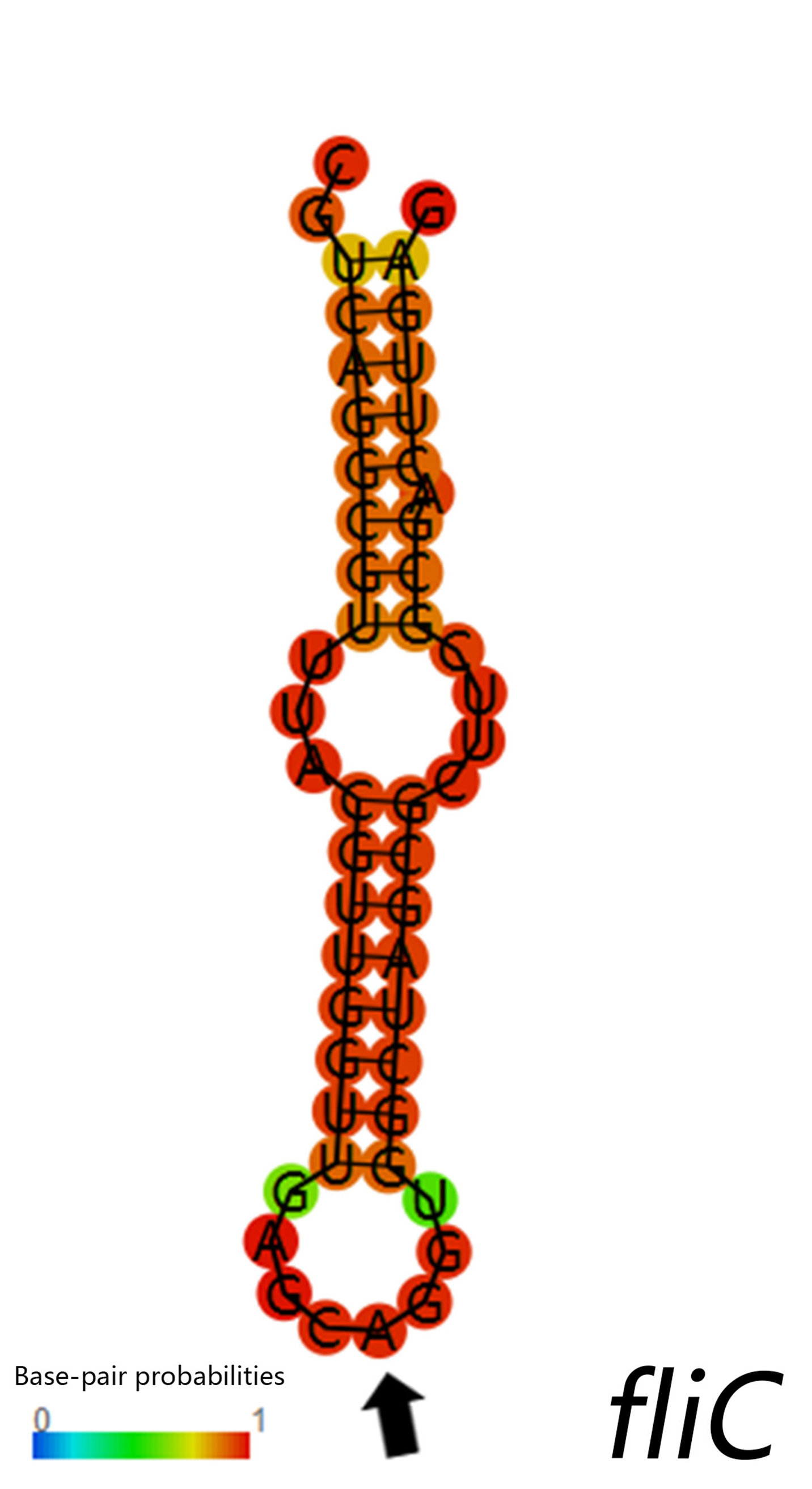

Supplement: S3 Fig — RNA secondary structure analysis (http://rna.tbi.univie.ac.at/) showed that the edited site is embedded within a loop (see arrow). (TIF) [file ppat.1008740.s003.tif]

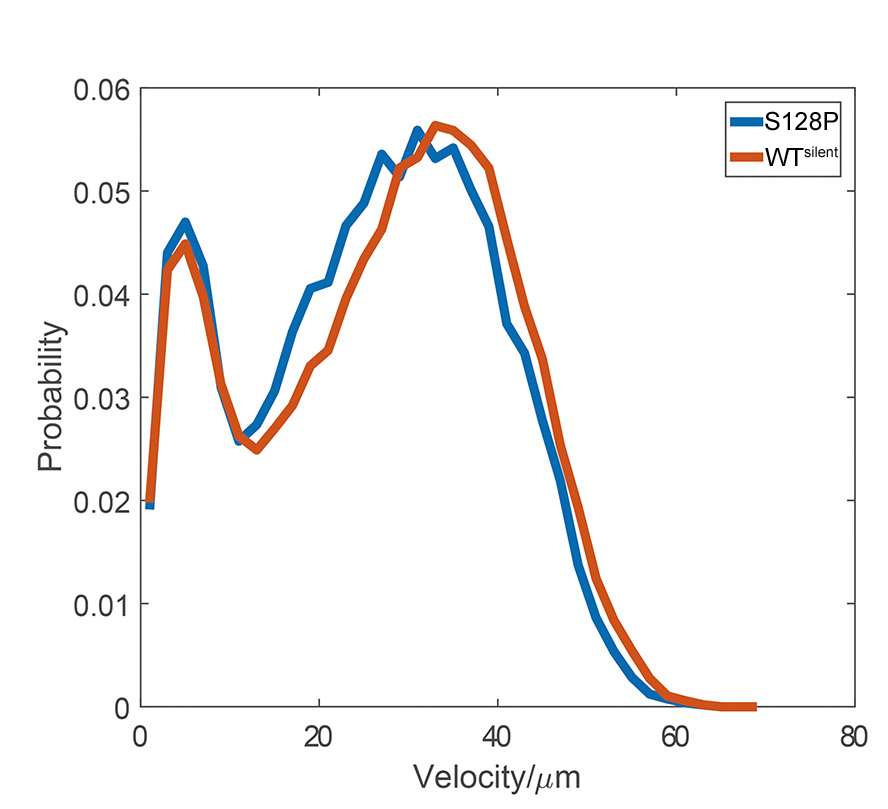

Supplement: S4 Fig — Bacterial cell velocity was randomly traced for 575 and 721 bacterial cells from S128P and WTsilent, respectively. (TIF) [file ppat.1008740.s004.tif]

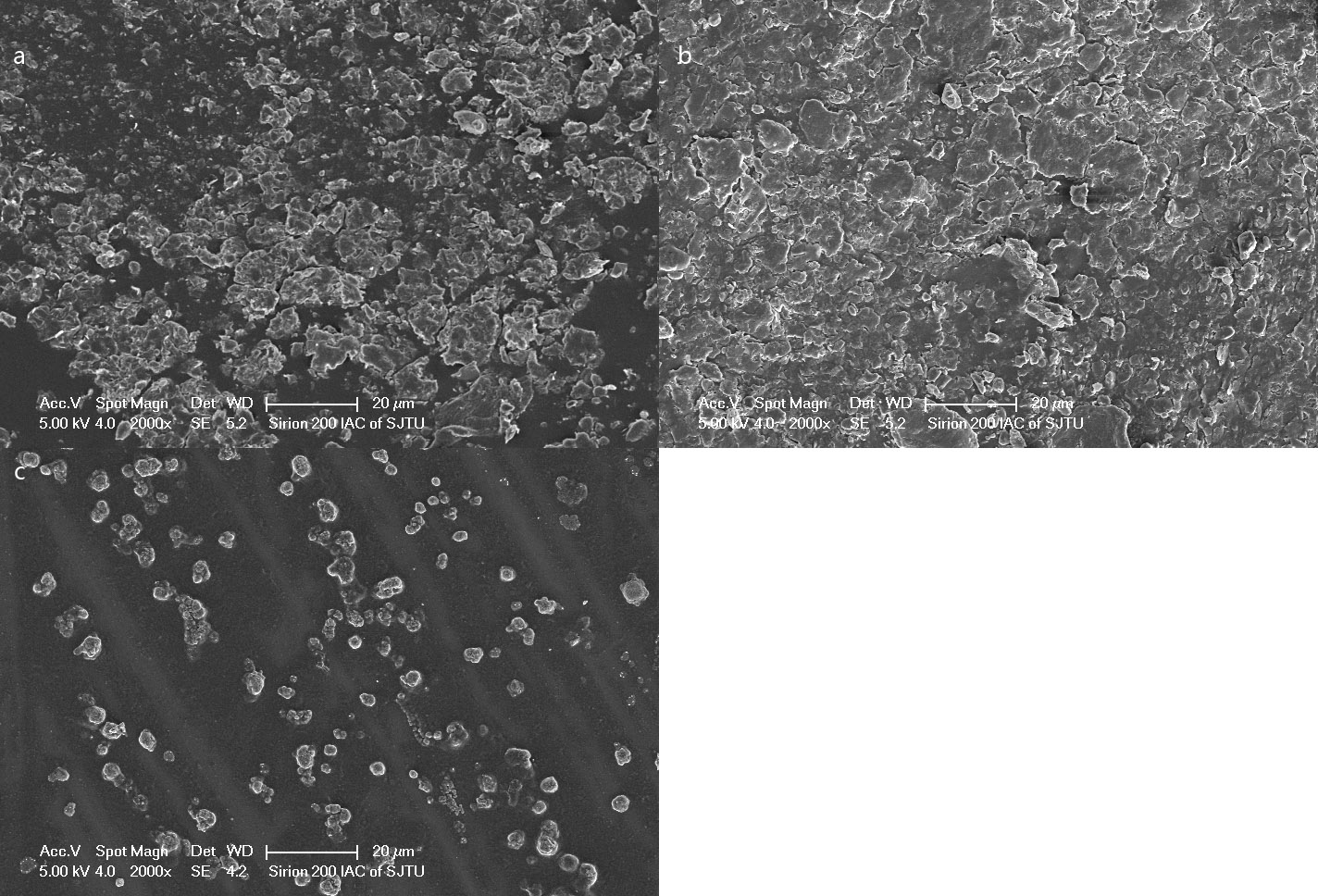

Supplement: S5 Fig — Panels: (a) WTsilent; (b) S128P mutant; and (c) Δ fliC mutant. (TIF) [file ppat.1008740.s005.tif]

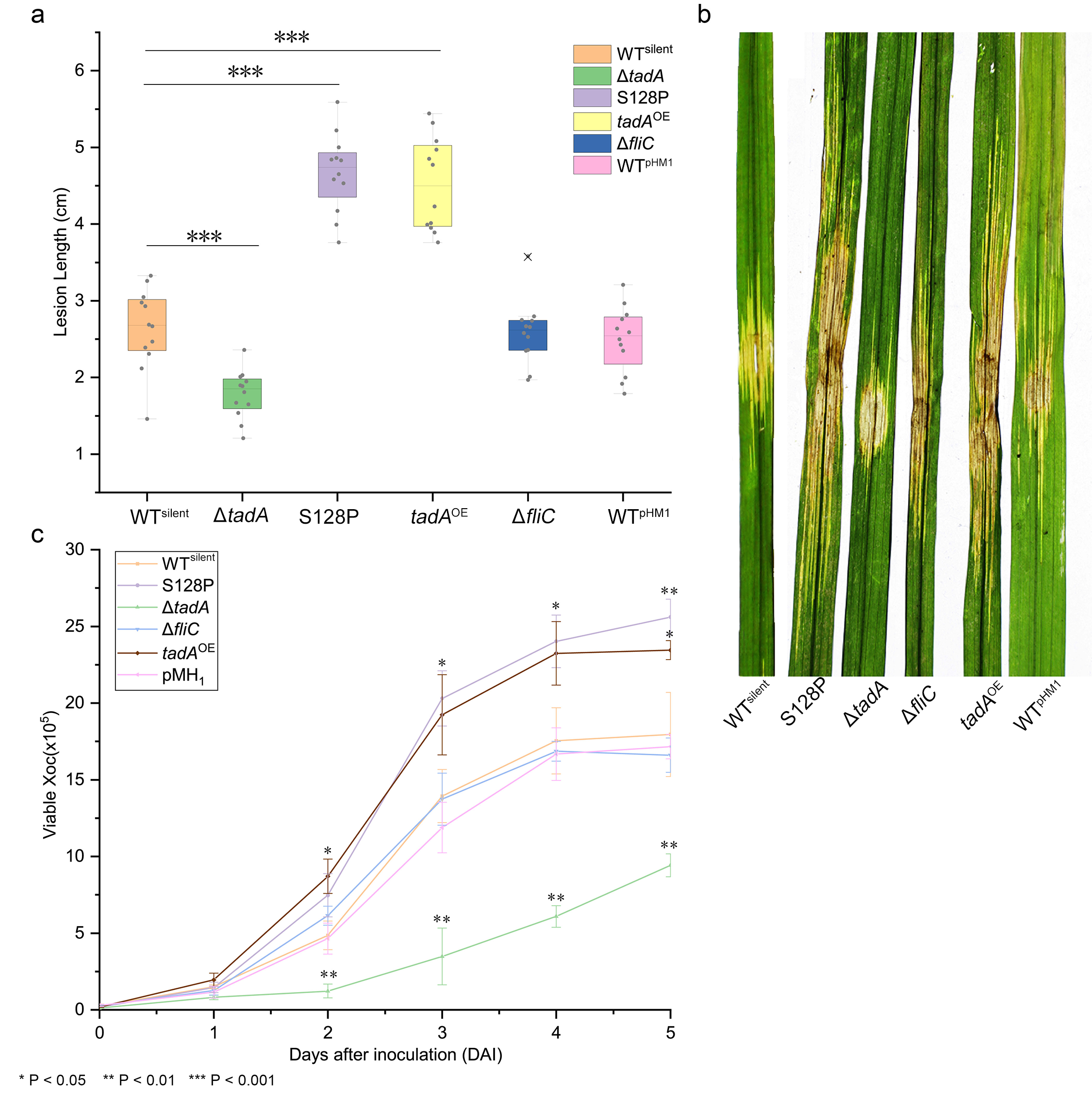

Supplement: S6 Fig — Virulence was assessed by inoculating six-week-old susceptible rice plants. (a) Twelve leaves were inoculated per strain with needleless syringes, and lesion lengths were evaluated 14 days after inoculation. Results indicate means ± SD; asterisks (***) show significant differences between mutants and WTsilent (P < 0.001; ANOVA with Dunnett’s multiple test post-hoc correction compared with WTsilent). (b) Symptoms on rice leaves inoculated with Xoc WTsilent, S128P mutant, ΔtadA, tadAOE and WTpHM1. (c) Number of viable WTsilent, S128P, Δ tadA, tadAOE and WTpHM1 cells in planta. Population dynamics of bacterial strains in infected leaves (means ± SD). Infiltrated regions were examined in three independent experiments (three leaves each from three independent experiments) for each strain of Xoc, *, P < 0.01; **, P < 0.001. (TIF) [file ppat.1008740.s006.tif]

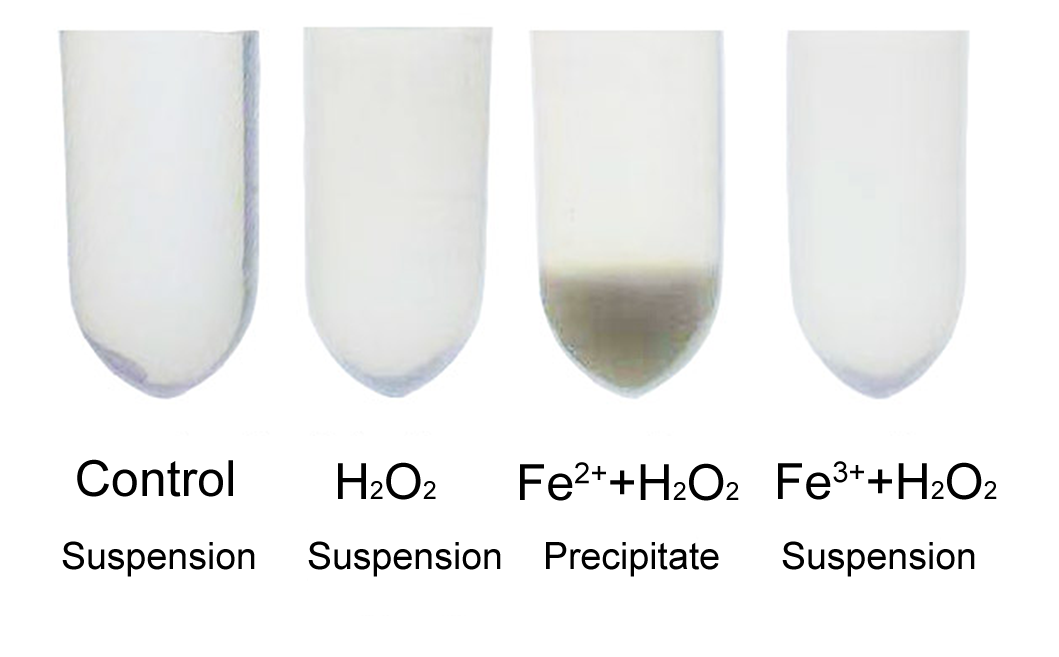

Supplement: S7 Fig — Precipitates were products of the Fenton reaction. (TIF) [file ppat.1008740.s007.tif]

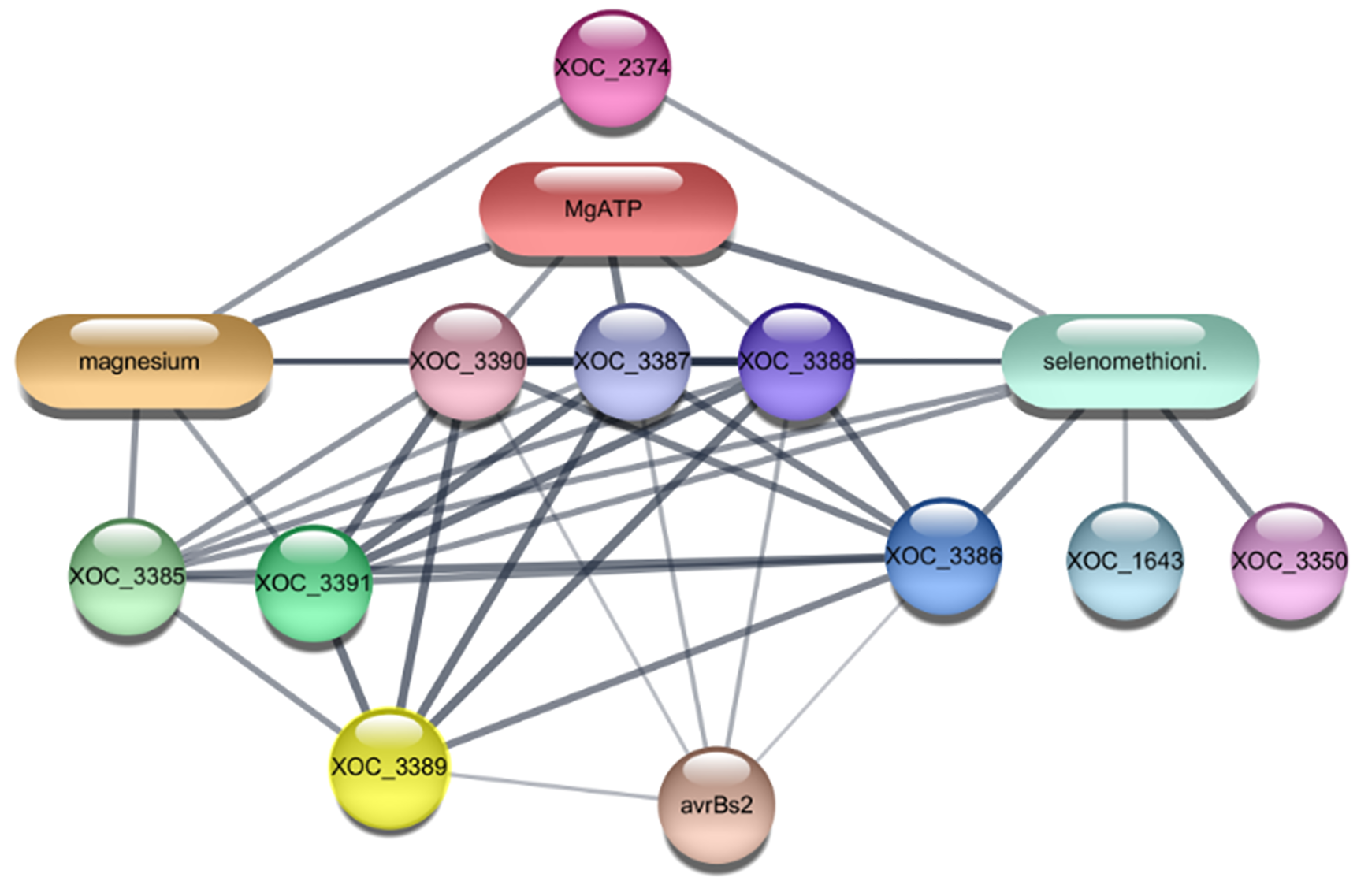

Supplement: S8 Fig — Line thickness correlates with the evidence supporting the interactions among proteins. (TIF) [file ppat.1008740.s008.tif]

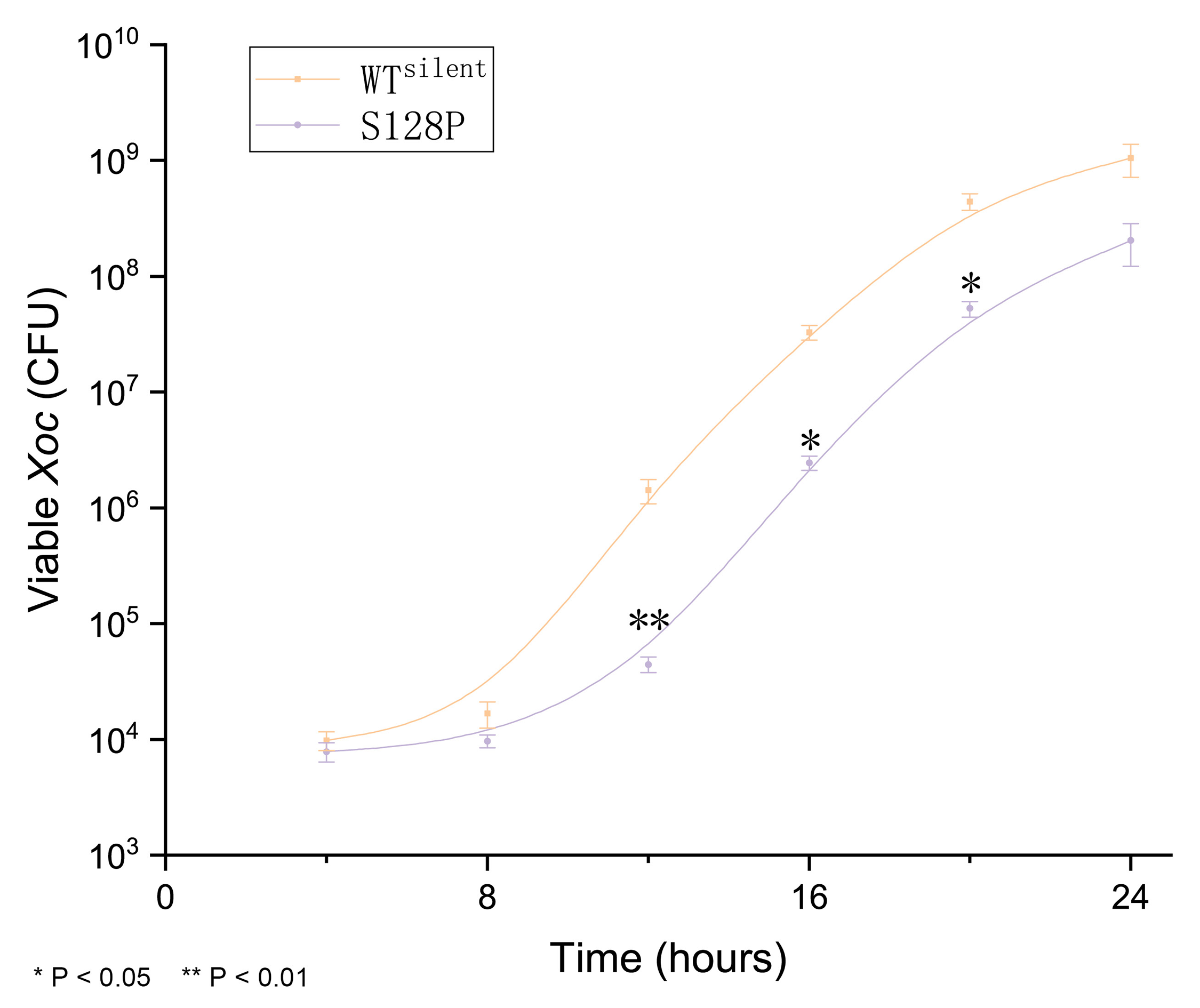

Supplement: S9 Fig — (TIF) [file ppat.1008740.s009.tif]

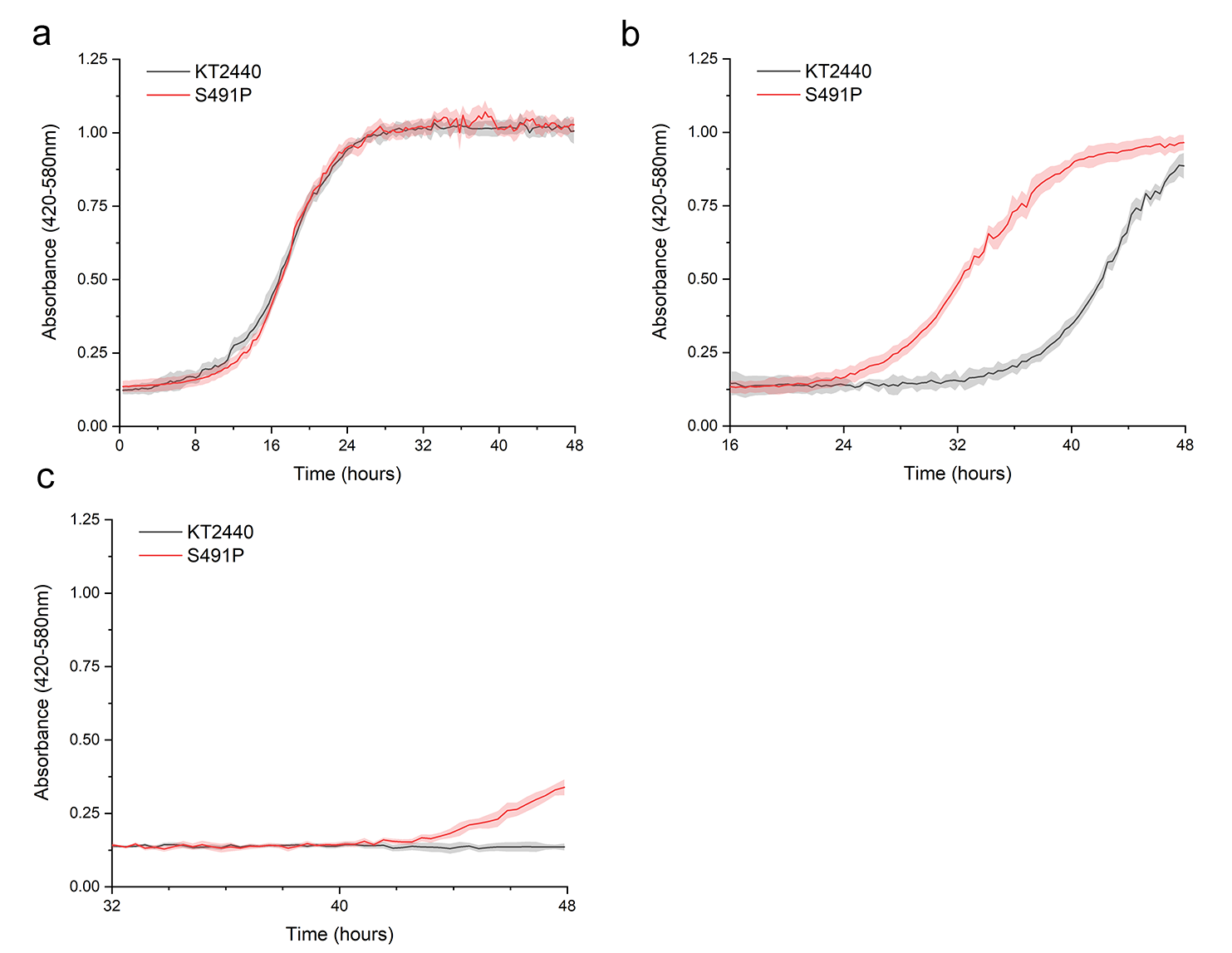

Supplement: S10 Fig — The strains were grown in quadruplicate to mid-exponential phase in NB, diluted to OD600 = 0.1, transferred to fresh NB and placed in a Bioscreen C apparatus at 28°C to monitor growth. Panels: (a) growth of P. putida KT2440 and S491P mutant in NB; (b) growth in NB supplemented with 2 mM H2O2; and (c) growth in NB supplemented with 4 mM H2O2. Data points represent mean OD values. (TIF) [file ppat.1008740.s010.tif]

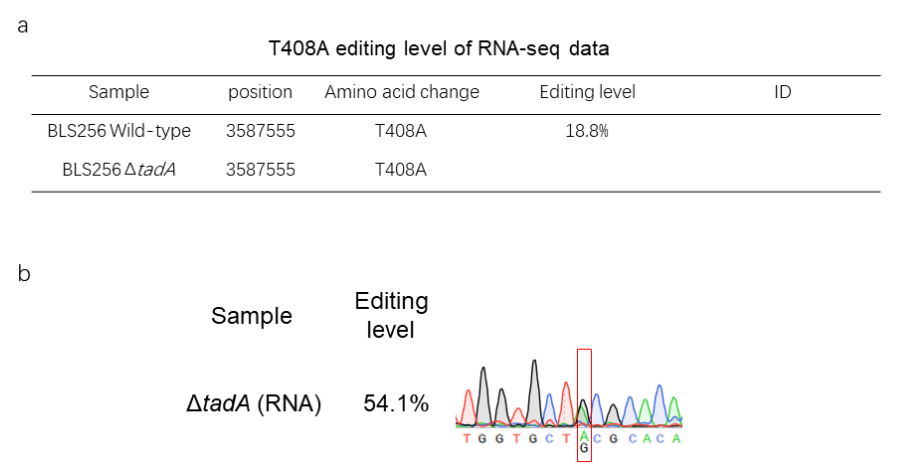

Supplement: S11 Fig — RNA-seq and Sanger sequencing results from T408A editing event in XOC_3486: (a) editing level from RNA-seq; (b) editing level from Sanger sequencing. (TIF) [file ppat.1008740.s011.tif]
