# Supplementary material for: A-to-I RNA editing in bacteria increases pathogenicity and tolerance to oxidative stress
Source: PLoS Pathog. 2020 Aug 21;16(8):e1008740. doi: 10.1371/journal.ppat.1008740 (PMC7467310; doi:10.1371/journal.ppat.1008740)
Supplement: S5 Table — (DOCX) [file ppat.1008740.s018.docx]

Table S5 Strains and plasmids used in this study.

| Strain or plasmid | Relevant characteristics | Source or reference |
| --- | --- | --- |
| Strains | | |
| *Escherichia coli* | | |
| DH5α | F^-^ Φ80d lacZΔM15Δ(lacZYA-argF) U169 *recA1 endA1, hsdR17*(r_k_^-^,m_k_^+^) *phoA supE44*λ^-^ *thi-1 gyrA96 relA1* |  |
| S17-1(λ pir) | λ lysogenic S17-1 derivative producing π protein for replication of plasmids carrying *oriR6K*; *recAprohsdR*RP4-2-Tc::Mu-Km::Tn7 λ^-^ pir | [1] |
| *Xanthomonas oryzae* pv. *oryzicola* | | |
| BLS256 | Wild-type | [2] |
| WT^silent^ | BLS256 containing a synonymous mutation in *fliC* that blocks A-to-I RNA editing at amino acid 128 | This study |
| S128P | BLS256 containing a mutation that changes the serine to proline at amino acid 128 in FliC | This study |
| Δ*tadA* | *tadA* knock-out mutant of BLS256 | This study |
|  |  |  |
| Δ*fliC* | *fliC* knock-out mutant of BLS256 | This study |
| *Δ3386* | *XOC_3386* knock-out mutant of BLS256 | This study |
| *tadA*^OE^ | BLS256 harboring pHM1::*tadA*, Sp^R^ | This study |
| WT^pHM1^ | BLS256 harboring empty pHM1, Sp^R^ | This study |
| WT^silent^-gfp | WT^silent^ harboring pHM1::*gfp*, Sp^R^ | This study |
| S128P-gfp | S128P harboring pHM1::*gfp*, Sp^R^ | This study |
| Δ*fliC-*gfp | Δ*fliC* harboring pHM1::*gfp*, Sp^R^ | This study |
| His-BLS256 | BLS256 containing a 6X-His-tag prior to the stop codon of *tadA* | This study |
| *Pseudomonas putida* | | |
| KT2440 | Wild-type | [3] |
| S491P | KT2440 containing a serine to proline mutation at amino acid residue 491 in FliC | This study |
| Plasmids | | |
| pKMS1 | Km^R^; R6K-based suicide vector; requires the *pir*-encoded π protein for replication | [4] |
| pHM1 | Sp^R^, *Mob*(*p*)*, IncW, Mob*^+^*, LaclP*^+^, PK2 replicon, cosmid | [5] |
| pKMS1::S128P | Used to introduce an A-to-G point mutation in BLS256 *fliC*; results in the S128P mutation; used for homologous recombination, Km^R^ | This study |
| pKMS1::Δ*tadA* | *tadA* knock-out cassette in pKMS1, Km^R^ | This study |
| pKMS1::Δ*fliC* | *fliC* knock-out cassette in pKMS1, Km^R^ | This study |
| pKMS1::Δ3387 | *XOC_3387* knock-out cassette in pKMS1, Km^R^ | This study |
| pKMS1::S491P | Used to introduce an A-to-G point mutation in *P. putida* KT2440 *fliC*; results in the S491P mutation; used for homologous recombination, Km^R^ | This study |
| pHM1::*tadA* | 529-bp *Sal*I/*Hin*dIII fragment containing *tadA* cloned in pHM1, Sp^R^ | This study |
| pHM1::*gfp* | 946-bp *Hin*d III/*Eco*R I fragment containing *gfp* cloned in pHM1, Sp^R^ | [6] |

a Sp^R^, spectinomycin resistance; Km^R^, kanamycin resistance.

**References**

1. Simon R, Priefer U, Pühler A. A broad host range mobilization system for in vivo genetic engineering: transposon mutagenesis in gram negative bacteria. Nat Biotechnol. 1983;1(9):784-91.

2. Bogdanove AJ, Koebnik R, Lu H, Furutani A, Angiuoli SV, Patil PB, et al. Two new complete genome sequences offer insight into host and tissue specificity of plant pathogenic *Xanthomonas* spp. J Bacteriol. 2011;193(19):5450-64.

3. Nelson KE, Weinel C, Paulsen IT, Dodson RJ, Hilbert H, Dos Santos VM, et al. Complete genome sequence and comparative analysis of the metabolically versatile *Pseudomonas putida* KT2440. Environ Microbiol. 2002;4(12):799-808.

4. Li Y-R, Zou H-S, Che Y-Z, Cui Y-P, Guo W, Zou L-F, et al. A novel regulatory role of HrpD6 in regulating hrp-hrc-hpa genes in *Xanthomonas oryzae* pv. *oryzicola*. Mol Plant Microbe Interact. 2011;24(9):1086-101.

5. Innes RW, Hirose MA, Kuempel PL. Induction of nitrogen-fixing nodules on clover requires only 32 kilobase pairs of DNA from the *Rhizobium trifolii* symbiosis plasmid. J Bacteriol. 1988;170(9):3793-802.

6. Li Y, Xiao Y, Zou L, Zou H, Chen G. Identification of HrpX regulon genes in *Xanthomonas oryzae* pv. *oryzicola* using a GFP visualization technique. Arch Microbiol. 2012;194(4):281-91.
