# Supplementary material for: A-to-I RNA editing in bacteria increases pathogenicity and tolerance to oxidative stress
Source: PLoS Pathog. 2020 Aug 21;16(8):e1008740. doi: 10.1371/journal.ppat.1008740 (PMC7467310; doi:10.1371/journal.ppat.1008740)
Supplement: S6 Table — (DOCX) [file ppat.1008740.s019.docx]

Table S6. Primers used in this study

| Application | | Primers | Sequence(5’ to 3’) |
| --- | --- | --- | --- |
| S128P point mutation | Amplification of BLS256 *fliC* | BLS *fliC* F | GGATCCTCTTGGCGTAGTCGGTATCG(B) |
|  |  | BLS *fliC* R | GCTCTAGAGCTCAGCGTAACCTCAACAC(X) |
|  | S128P point mutation | S128P F | CGCTTCGCGATCGGTGGGCGAGTTGGTTGCA |
|  |  | S128P R | CCCACCGATCGCGAAGCGCTGAACTCCGAAG |
| WT^silent^ | S128 point synonymous mutation | Silent F | GCAAATGCAACCAACTCGAGCACCGATCGCGAA |
|  |  | Silent R | CTCGAGTTGGTTGCATTTGCGGACTGCACTGAC |
| Amplification to verify the point mutant occurrence | | Verify F | ATAGGTCACGCTCACGAGAT |
|  |  | Verify R | CTCTTGATACACAACGCTTACG |
| Amplification of cDNA to determine the *fliC* editing level | | Edit F | GGAGGTGAGCTGCTTGACTT |
|  |  | Edit R | GTCAGTGCAGTCCGCAAAT |
| Amplification of *tadA* deletion cassette | | *tadA* upstream F | AACTGCAGTTCCACATCCATAGCGGTATC(P) |
|  |  | *tadA* upstream R | CCCCCGGGGACCAATTACTTCAGAGCCAAG(S) |
|  |  | *tadA* downstream F | CCCCCGGGGTGGCTGGCGATATTGAAGTT(S) |
|  |  | *tadA* downstream R | GCTCTAGAGGACGGGATGACGAGTATGAT(X) |
| Amplification of *fliC*  deletion cassette | | BLS *fliC* upstream F | CGGGATCCGGAATCGACCGCATTGAAGAC(B) |
|  |  | BLS *fliC* upstream R | CCCCCGGGTTAGCGGCAGCGATAGGTC(S) |
|  |  | BLS *fliC* downstream F | CCCCCGGGTACCGCTGCTGAGATGAATAAC(S) |
|  |  | BLS *fliC* downstream R | GCTCTAGAGCCGCCAACACCTATGAAG(X) |
| Amplification of *XOC_3386*  deletion cassette | | *XOC_3386* upstream F | AACTGCAGCACTCGTTCCTCAGGTAGC(P) |
|  |  | *XOC_3386* upstream R | CGGAATTCATTGCTGCGATGGCGATGG(E) |
|  |  | *XOC_3386* downstream F | CGGAATTCGTTGGTCACGCATTGAAGCA(E) |
|  |  | *XOC_3386* downstream R | GCTCTAGACATCGCCGCCTTGTTCTTG(X) |
| Amplification of *tadA* | | *tadA* F | GCGTCGACCAAGGTTCTCTGCCATATCG(Sa) |
|  |  | *tadA* R | CCAAGCTTTCAGTGAGGTGACGGTTG(H) |
| *tadA* His-tag introduced by overlap PCR | | *tadA*Ht upstream F | AACTGCAGTCAGTGAGGTGACGGTTGC(P) |
|  |  | *tadA*Ht upstream R | TCAGTGATGGTGATGGTGATGCGGCGCAAGTGGCGGCTTG |
|  |  | *tadA*Ht downstream F | CATCACCATCACCATCACTGAGCGACGCGGCCGCGGTATG |
|  |  | *tadA*Ht downstream R | GCTCTAGAACGCCAGCAGATTCTCAGC(X) |
| S491P point mutation | Amplification of KT2440 *fliC* | KT *fliC* F | CGGGATCCTCAGCGAACAGCGACAGAG(B) |
|  |  | KT *fliC* R | GCTCTAGATGGCGACTTCAGGCAACAT(X) |
|  | S491P point mutation | S491P F | AGCCTTGGTGGTGCCCGGGTTCAGACCTACG |
|  |  | S491P R | CCGGGCACCACCAAGGCTGGTTTGGTTGCTG |
| Amplification to verify the point mutant occurrence of KT2440 | | KT Verify F | CGACAGGATAGCGGTGGAA |
|  |  | KT Verify R | GGCGACTTCAGGCAACATC |
| Amplification of cDNA to determine the *fliC* editing level of KT2440 | | KT Edit F | GATGGAGGCTGCGATGCTA |
|  |  | KT Edit R | CAATGGTTCTGGCGTGACTG |
| qPCR analysis on *XOC_3386* | | *XOC_3386* F | TGGATGCCAGCCAGGAATC |
|  |  | *XOC_3386* R | GTGTGACCGAATGCGTAGC |
| qPCR analysis on *XOC_3387* | | *XOC_3387* F | TTACGCTGGAAACGCTTGG |
|  |  | *XOC_3387* R | CGAAGGCACGCAGTTGTT |
| qPCR analysis on *XOC_3388* | | *XOC_3388* F | CATCCGCAACTGCATCTCAA |
|  |  | *XOC_3388* R | GACGAACACACAACGACCTT |
| qPCR analysis on *XOC_3389* | | *XOC_3389* F | CGACAGCAACAGCAGCAAT |
|  |  | *XOC_3389* R | GCACCACCAGATAGACCAGAT |
| qPCR analysis on *XOC_3390* | | *XOC_3390* F | CGATCACCGCTGCGTTATTAC |
|  |  | *XOC_3390* R | CGATCCAGATCCACGCCTTC |
| qPCR analysis on *rpoD* as internal control | | *rpoD* F | CGACAACACCACCAACATCAATC |
|  |  | *rpoD* R | GCTTACCGACCTCTTCCAACG |

a The sequences underline are the introduced restriction sites of enzymes indicated in parentheses. (B=BamHI; E=EcoRI; H=HindIII; S=SmaI; Sa=SalI; X=XbaI)

b The nucleobases in red marked as the artificial point mutant introduced.

c The nucleobases in blue marked as the artificial 6-Hig-tag introduced, and the next TGA is *tadA* stop codon.
